# Testing the utility of DNA barcodes and a preliminary phylogenetic framework for Chinese freshwater mussels (Bivalvia: Unionidae) from the middle and lower Yangtze River

**DOI:** 10.1371/journal.pone.0200956

**Published:** 2018-08-08

**Authors:** Rui-Wen Wu, Yi-Tong Liu, Sa Wang, Xiong-Jun Liu, David T. Zanatta, Kevin J. Roe, Xue-Lin Song, Chang-Ting An, Xiao-Ping Wu

**Affiliations:** 1 School of Life Sciences, Nanchang University, Nanchang, Jiangxi, People’s Republic of China; 2 School of Resources Environmental & Chemical Engineering, Nanchang University, Nanchang, Jiangxi, People’s Republic of China; 3 Biology Department, Central Michigan University, Mount Pleasant, Michigan, United States of America; 4 Department of Natural Resource Ecology and Management, Iowa State University, Ames, Iowa, United States of America; 5 Institute of Hydrobiology, Chinese Academy of Sciences, Wuhan, Hubei, People’s Republic of China; 6 Center for Watershed Ecology, Institute of Life Science, Nanchang University, Nanchang, Jiangxi, People’s Republic of China; National Cheng Kung University, TAIWAN

## Abstract

The middle and lower portions of the Yangtze River basin is the most species-rich region for freshwater mussels in Asia. The management and conservation of the taxa in this region has been greatly hampered by the lack of a well-developed phylogeny and species-level taxonomic framework. In this study, we tested the utility of two mitochondrial genes commonly used as DNA barcodes: the first subunit of the cytochrome oxidase c gene (COI) and the first subunit of the NADH dehydrogenase gene (ND1) for 34 putative species representing 15 genera, and also generated phylogenetic hypotheses for Chinese unionids based on the combined dataset of the two mitochondrial genes. The results showed that both loci performed well as barcodes for species identification, but the ND1 sequences provided better resolution when compared to COI. Based on the two-locus dataset, Bayesian Inference (BI) and Maximum Likelihood (ML) phylogenetic analyses indicated 3 of the 15 genera of Chinese freshwater mussels examined were polyphyletic. Additionally, the analyses placed the 15 genera into 3 subfamilies: Unioninae (*Aculamprotula*, *Cuneopsis*, *Nodularia* and *Schistodesmus*), Gonideninae (*Lamprotula*, *Solenaia* and *Ptychorhychus*) and Anodontinae (*Cristaria*, *Arconaia*, *Acuticosta*, *Lanceolaria*, *Anemina* and *Sinoanodonta*). Our results contradict previous taxonomic classification that placed the genera *Arconaia*, *Acuticosta* and *Lanceolaria* in the Unioninae. This study represents one of the first attempts to develop a molecular phylogenetic framework for the Chinese members of the Unionidae and will provide a basis for future research on the evolution, ecology, and conservation of Chinese freshwater mussels.

## Introduction

Freshwater mussels of the order Unionida comprise a significant proportion of the benthic biomass, and can have a significant influence on the community structure of the benthos [1]. As filter feeders, the Unionida provide important ecological functions and ecosystem services [2]. Current estimates indicate there are 840 species of freshwater mussels in the world, belonging to 6 families and 180 genera. Among them, the Unionidae is by far the most species-rich family with 620 species in 142 genera, and is widely distributed in North America, Eurasia, Central America, Africa and Southeast Asia [3–4].

Freshwater mussels are considered to be some of the most threatened freshwater taxa in the world [5–6]. The middle and lower portions of the Yangtze River basin in China is the most species-rich regions for freshwater mussels in Asia [3, 7], and includes a number of endemic species [8–9]. According to the surveys of species diversity conducted over the last ten years, there are up to 15 genera of Unionidae represented in the middle and lower reaches of the Yangtze River [9–10]. However, in the past two decades, anthropogenic stressors, including habitat destruction and degradation, commercial exploitation, and water pollution have had negative impacts on the survival and reproduction for many mussel species, and the imperilment of a number or endemic populations in the region [11–12].

Understanding phylogenetic diversity is crucial for conservation prioritization of freshwater mussels, but until recently, taxonomic and phylogenetic work in China has lagged relative to North America and Europe. The first and most important classification of the Chinese unionid fauna was attempted by Heude beginning in 1875 [13–22]. His collected works on Chinese taxa resulted in the classification of 140 species based on shell morphology. Over the past century, there has been substantial disagreement on the validity of species and the taxonomy of this group [8, 23–24]. Since 1949, a number of faunal investigations were conducted that improved the accuracy of species ranges [25–30], but the classifications were still based on shell morphology alone. The inclusion of anatomical characters, such as the morphology of the marsupium and larval type [31–35], did improve the classification of Chinese taxa; however, these advancements were largely restricted to the genus and species-level classification of Chinese Unionidae.

Accurate identification of biological diversity is an important component in the conservation of species. One of the greatest barriers to the conservation of endangered species is our lack of knowledge of their existence. Application of molecular genetic tools has resulted in a dramatic increase in the amount of biodiversity recognized to date [36]. Molecular genetic markers have the potential to provide more objective and accurate characters for improving our understanding of the systematics and taxonomy, evolutionary history and genetic diversity of Chinese freshwater mussels [37]. The number of studies examining the phylogenetic relationships of Chinese freshwater mussels based on molecular data has increased recently [38–49]; however, most of the studies included only a limited number genera, species, and specimens. Other studies have attempted to construct a phylogenetic framework for Chinese mussels [50–51], but suffered due to limited taxon sampling; thus, an integrated phylogenetic framework for Chinese taxa is still lacking.

DNA barcoding technology has proven to be a reliable tool in species identification and phylogenetic analysis [52]. The first subunit of the cytochrome oxidase c gene (COI) and the first subunit of the NADH dehydrogenase gene (ND1) are widely used in phylogenetic analysis, taxonomic identification and identification of cryptic species [51–58], but information on the usefulness of DNA barcodes for Chinese mussels is largely lacking. There are only COI and/or ND1 sequences from a dozen Chinese mussel taxa in GenBank at this time, which severely hinders our understanding of phylogenetic diversity and monitoring of the fauna in China using environmental DNA (eDNA).

The middle and lower reaches of the Yangtze River in China represents one of the most-species rich regions for freshwater mussels on earth [7]. By sampling the tributary lakes and rivers in the middle and lower reaches of the Yangtze River, we were able to collect 34 putative species representing 15 genera of freshwater mussels. Our goals were to: (1) evaluate the efficacy of the mitochondrial COI and ND1 loci for DNA barcoding of Chinese freshwater mussels; and (2) begin to develop a modern phylogenetic framework for the Unionidae in China thereby placing more Chinese species into a global taxonomic classification.

## Materials and methods

### Ethics statement

All necessary permits were obtained for the described field studies from the Yangtze River Fishery Administration of China. The handling of mussels was conducted in accordance with the guidelines on the care and use of animals for scientific purposes set by the Institutional Animal Care and Use Committee (IACUC) of Nanchang University, Jiangxi, China.

### Taxon sampling

Unionids were collected between 2014 and 2017 from a selection of lakes and tributaries in the middle and lower reaches of the Yangtze River, including Dongting Lake (Hunan Province, N: 28.87.65° ~ 29.23°; E: 112.62° ~ 113.00°), Liangzi Lake (Hubei Province, N: 30.22°; E: 114.60°), Poyang Lake (Jiangxi Province, N: 29.03° ~ 29.17°; E: 116.20° ~ 116.26°), Xiannv Lake (Jiangxi Province, N: 27.72°; E: 114.80°), Gan River (Jiangxi Province, N: 28.57° ~ 28.86°; E: 115.57° ~ 116.00°), Tai Lake (Jiangsu Province, N: 31.24°; E: 120.23°) and Hongze Lake (Jiangsu Province, N: 33.22°; E: 118.68°). Field-collected specimens were taken to the laboratory and identified to species based on the shell morphology. At present, there are three authoritative publications on the classification of Chinese freshwater bivalves [8, 29, 59]. However, the taxonomy of the Asian unionid fauna is continuing to evolve as more studies are published [7, 41, 49]. For this study, we based our identification of freshwater mussels on these publications. In addition, we made use of the MusselP website [60], which provided a global taxonomic framework and pictures of type specimens, which greatly facilitated identifications. Gender for each individual was determined by gonad smear [61]. All Chinese species collected and examined are listed in Table 1. A small sample of foot tissue was removed from each specimen and preserved in 96% ethanol for later DNA extraction. Voucher specimens representing the species included in this study were deposited in the Nanchang University Museum of Biology.

**Table 1:** Freshwater mussel species used in this study. Haplotypes were estimated for ingroup taxa by species.

Taxon	Haplotype No.	COI	NDI	Collection Location	Reference
ANODONTINAE					
*Acuticosta chinensis* (Lea, 1868)	1	MG933696	MG933755	Poyang Lake, Gan River, China	This study
*A*.*chinensis*	2	KJ434469	DQ077897	Poyang Lake, Gan River, China	Ouyang et al., 2015 [51]; Unpublished
*A*.*chinensis*	3	KJ434471	KJ434535	Poyang Lake, Gan River, China	Ouyang et al., 2015 [51]
*A*.*chinensis*	4	KJ434472	KJ434536	Poyang Lake, Gan River, China	Ouyang et al., 2015 [51]
*A*.*chinensis*	5	KJ434475	KJ434537	Poyang Lake, Gan River, China	Ouyang et al., 2015 [51]
*A*.*chinensis*	6	n/a	KJ434540	Poyang Lake, Gan River, China	Ouyang et al., 2015 [51]
*A*.*chinensis*	7	n/a	KJ434541	Poyang Lake, Gan River, China	Ouyang et al., 2015 [51]
*Acuticosta ovata* (Simpson, 1900)	1	n/a	KJ434542	Poyang Lake, Gan River, China	Ouyang et al., 2015 [51]
*A*.*ovata*	2	n/a	KJ434543	Poyang Lake, Gan River, China	Ouyang et al., 2015 [51]
*Alasmidonta marginata* Say, 1818	1	AF156502	GU085335	USA	Graf & Foighil, 2000 [90]; Boyer et al., 2011 [53]
*Anemina angula* Tchang et al., 1965	1	MG933697	MG933756	Xiannv Lake, China	This study
*A*.*angula*	2	MG933698	n/a	Xianlv Lake, China	This study
*Anemina arcaeformis* (Heude, 1877)	1	MG933699	MG933757	Dongting Lake, China	This study
*A*.*arcaeformis*	2	MG933700	MG933758	Dongting Lake, China	This study
*A*.*arcaeformis*	3	MG933701	MG933759	Dongting Lake, China	This study
*A*.*arcaeformis*	4	MG933702	n/a	Dongting Lake, China	This study
*A*.*arcaeformis*	5	MG933703	n/a	Dongting Lake, China	This study
*Anemina globosula* (Heude, 1878)	1	MG933704	MG933760	Dongting Lake, China	This study
*A*.*globosula*	2	MG933705	n/a	Dongting Lake, China	This study
*A*.*globosula*	3	MG933706	n/a	Dongting Lake, China	This study
*Arconaia lanceolata* (Lea, 1856)	1	MG933707	MG933761	Tai Lake, China	This study
*A*.*lanceolata*	2	n/a	MG933762	Tai Lake, China	This study
*A*.*lanceolata*	3	n/a	MG933763	Tai Lake, China	This study
*Anodonta anatina* (Linnaeus, 1758)	1	NC_022803	NC_022803	USA	Soroka, 2015 [91]
*Anodonta cygnea* (Linnaeus, 1758)	1	NC_036488	NC_036488	USA	Soroka & Burzyński, 2017 [92]
*Cristaria plicata* (Leach, 1814)	1	MG933708	MG933764	Liangzi Lake, China	This study
*C*.*plicata*	2	EU698939	MG933765	Liangzi Lake, China	Unpublished; This study
*C*.*plicata*	3	EU698941	n/a	Poyang Lake, China	Unpublished
*C*.*plicata*	4	EU698944	n/a	Tai Lake, China	Unpublished
*C*.*plicata*	5	EU698945	n/a	Qiantang River, China	Unpublished
*C*.*plicata*	6	EU698946	n/a	Qiantang River, China	Unpublished
*C*.*plicata*	7	EU698947	n/a	Qiantang River, China	Unpublished
*C*.*plicata*	8	EU698948	n/a	Qiantang River, China	Unpublished
*C*.*plicata*	9	EU698949	n/a	Qiantang River, China	Unpublished
*C*.*plicata*	10	GQ451860	n/a	Unknown	Unpublished
*Lanceolaria gladiola* (Heude, 1877)	1	MG933720	MG933780	Poyang Lake, China	This study
*Lanceolaria grayii* (Lea, 1834)	1	MG933721	NC_026686	Poyang Lake, China	This study; Wang et al., 2016 [93]
*L*. *grayii*	2	KJ434525	n/a	Poyang Lake, Gan River, China	Ouyang et al., 2015 [51]
*Lanceolaria triformis* (Heude, 1877)	1	MG933722	MG933781	Gan River, China	This study
*L*.*triformis*	2	MG933723	MG933782	Gan River, China	This study
*L*.*triformis*	3	MG933724	MG933783	Gan River, China	This study
*Lanceolaria eucylindrica* Lin, 1962	1	n/a	MG933777	Gan River, China	This study
*L*.*eucylindrica*	2	n/a	MG933778	Gan River, China	This study
*L*.*eucylindrica*	3	n/a	MG933779	Gan River, China	This study
*Lasmigona compressa* (Lea, 1829)	1	HM856638	HM856638	USA	Breton et al., 2011 [94]
*Pyganodon grandis* (Say, 1829)	1	KM262551	MG199751	UAS	Krebs et al., 2015 [95]; Smith et al., 2017 [96]
*Pyganodon cataracta* (Say, 1817)	1	JX101491	EF446102	USA	Hoeh et al., 2012 [97]; Kneeland & Rhymer, 2007 [98]
*Sinanodonta lucida* (Heude, 1877)	1	MG933735	MG933792	Poyang Lake, China	This study
*S*.*lucida*	2	n/a	MG933793	Poyang Lake, China	This study
*Sinanodonta woodiana* (Lea, 1834)	1	MG933736	MG933794	Gan River, China	This study
*S*.*woodiana*	2	MG933737	MG933795	Gan River, China	This study
*S*.*woodiana*	3	MG933738	MG933796	Gan River, China	This study
*S*.*woodiana*	4	MG933739	n/a	Gan River, China	This study
*Sinanodonta elliptica* (Heude, 1878)	1	MG933740	MG933797	Gan River, China	This study
*S*. *elliptica*	2	MG933741	MG933798	Gan River, China	This study
*S*. *elliptica*	3	MG933742	MG933799	Gan River, China	This study
*S*. *elliptica*	4	n/a	MG933800	Gan River, China	This study
*Strophitus undulatus* (Say, 1817)		AF231740	EF446100	USA	Bogan & Hoeh, 2000 [99]; Kneeland & Rhymer, 2015 [54]
UNIONINAE					
*Aculamprotula coreana* (Martens, 1886)	1	NC_026035	NC_026035	Korea	Unpublished
*Aculamprotula fibrosa* (Heude, 1877)	1	MG933687	MG933746	Poyang Lake, China	This study
*Aculamprotula scripta* (Heude, 1875)	1	MG933688	MG933747	Dongting Lake, China	This study
*A*.*scripta*	2	MG933689	MG933748	Dongting Lake, China	This study
*A*.*scripta*	3	n/a	MG933749	Dongting Lake, China	This study
*Aculamprotula tientsinensis* (Crosse & Debeaux, 1863)	1	MG933690	MG933750	Poyang Lake, China	This study
*A*.*tientsinensis*	2	n/a	MG933751	Poyang Lake, China	This study
*Aculamprotula tortuosa* (Lea, 1865)	1	MG933691	MG933752	Poyang Lake, China	This study
*A*.*tortuosa*	2	MG933692	MG933753	Poyang Lake, China	This study
*A*.*tortuosa*	3	MG933693	MG933754	Gan River, China	This study
*A*.*tortuosa*	4	MG933694	n/a	Gan River, China	This study
*A*.*tortuosa*	5	MG933695	n/a	Gan River, China	This study
*A*.*tortuosa*	6	NC_021404	n/a	Gan River, China	Wang et al., 2014 [42]
*Cuneopsis celtiformis* (Heude, 1874)	1	MG933709	MG933766	Poyang Lake, China	This study
*C*.*celtiformis*	2	KJ434491	KJ434557	Poyang Lake, Gan River, China	Ouyang et al., 2015 [51]
*C*.*celtiformis*	3	KJ434492	n/a	Poyang Lake, Gan River, China	Ouyang et al., 2015 [51]
*Cuneopsis heudei* (Heude, 1874)	1	MG933710	MG933767	Gan River, China	This study
*C*.*heudei*	2	KJ434494	KJ434560	Poyang Lake, Gan River, China	Ouyang et al., 2015 [51]
*Cuneopsis pisciculus* (Heude, 1874)	1	MG933711	MG933768	Gan River, China	This study
*C*.*pisciculus*	2	KJ434496	KJ434561	Poyang Lake, Gan River, China	Ouyang et al., 2015 [51]
*C*.*pisciculus*	3	KJ434497	KJ434562	Poyang Lake, Gan River, China	Ouyang et al., 2015 [51]
*C*.*pisciculus*	4	KJ434498	KJ434563	Poyang Lake, Gan River, China	Ouyang et al., 2015 [51]
*C*.*pisciculus*	5	n/a	KJ434564	Poyang Lake, Gan River, China	Ouyang et al., 2015 [51]
*Cuneopsis rufescens* (Heude, 1874)	1	MG933712	KJ434566	Poyang Lake, Gan River, China	This study; Ouyang et al., 2015 [51]
*C*.*rufescens*	2	KX822640	n/a	Poyang Lake, China	Lopes-Lima et al., 2017 [49]
*Lepidodesma languilati* (Heude, 1874)	1	MG933725	MG933784	Poyang Lake, China	This study
*L*.*languilati*	2	MG933726	MG933785	Poyang Lake, China	This study
*L*.*languilati*	3	MG933727	n/a	Poyang Lake, China	This study
*Nodularia douglasiae* (Griffith & Pidgeon, 1833)	1	MG933728	MG933786	Hongze Lake, China	This study
*N*.*douglasiae*	2	KJ434521	n/a	Poyang Lake, Gan River, China	Ouyang et al., 2015 [51]
*N*.*douglasiae*	3	KJ434522	n/a	Poyang Lake, Gan River, China	Ouyang et al., 2015 [51]
*N*.*douglasiae*	4	GQ451862	n/a	Unknown	Unpublished
*N*.*douglasiae*	5	GQ451863	n/a	Unknown	Unpublished
*Schistodesmus lampreyanus* (Baird & Adams, 1867)	1	MG933731	MG933789	Poyang Lake, China	This study
*S*.*lampreyanus*	2	MG933732	MG933790	Poyang Lake, China	This study
*S*.*lampreyanus*	3	KJ434509	n/a	Poyang Lake, Gan River, China	Ouyang et al., 2015 [51]
*S*.*lampreyanus*	4	MG933733	n/a	Poyang Lake, China	This study
*Schistodesmus spinosus* (Simpson, 1900)		MG933734	MG933791	Dongting Lake, China	This study
*Unio crassus* Retzius, 1788	1	KJ525919	KJ525928	France	Unpublished
*Unio pictorum* (Linnaeus, 1758)	1	EU548057	NC_015310	Poland	Soroka, 2010 [100]; Soroka & Burzynski, 2010 [101]
GONIDEINAE					
*Gonidea angulata* (Lea, 1838)	1	DQ272373	AY655099	USA	Gustafson & Iwamoto, 2005 [102]; Campbell et al., 2005 [103]
*Lamprotula caveata* (Heude, 1877)	1	MG933713	MG933769	Gan River, China	This study
*L*.*caveata*	2	MG933714	MG933770	Gan River, China	This study
*L*.*caveata*	3	MG933715	MG933771	Gan River, China	This study
*L*.*caveata*	4	MG933716	n/a	Gan River, China	This study
*Lamprotula leaii* (Griffith & Pidgeon, 1833)	1	MG933717	MG933772	Gan River, China	This study
*L*.*leaii*	2	MG933718	MG933773	Gan River, China	This study
*L*.*leaii*	3	MG933719	MG933774	Poyang Lake, China	This study
*L*.*leaii*	4	n/a	MG933775	Poyang Lake, China	This study
*L*.*leaii*	5	n/a	MG933776	Poyang Lake, China	This study
*Potomida littoralis* (Cuvier, 1798)	1	NC_030073	NC_030073	France	Froufe et al., 2016 [104]
*Ptychorhynchus pfisteri* (Heude, 1874)	1	MG933729	MG933787	Dongting Lake, China	This study
*P*.*pfisteri*	2	MG933730	MG933788	Dongting Lake, China	This study
*Sinohyriopsis cumingii* (Lea, 1852)	1	MG933743	MG933801	Tai Lake, China	This study
*S*.*cumingii*	2	KJ434500	MG933802	Poyang Lake, Gan River, China	Ouyang et al., 2015 [51]; This study
*S*.*cumingii*	3	KJ434501	MG933803	Poyang Lake, Gan River, China	Ouyang et al., 2015 [51]; This study
*S*.*cumingii*	4	KJ434502	n/a	Poyang Lake, Gan River, China	Ouyang et al., 2015 [51]
*Solenaia carinata* (Heude, 1877)	1	MG933744	MG933804	Gang River, China	This study
*Solenaia oleivora* (Heude, 1877)	1	KJ434511	KJ434581	Poyang Lake, Gan River, China	Ouyang et al., 2015 [51]
*S*.*oleivora*	2	KJ434513	KJ434583	Poyang Lake, Gan River, China	Ouyang et al., 2015 [51]
*S*.*oleivora*	3	KJ434514	KJ434586	Poyang Lake, Gan River, China	Ouyang et al., 2015 [51]
*S*.*oleivora*	4	KJ434516	n/a	Poyang Lake, Gan River, China	Ouyang et al., 2015 [51]
*Solenaia rivularis* (Heude, 1877)	1	MG933745	MG933805	Gan River, China	This study
*S*.*rivularis*	2	KJ434528	KJ434588	Poyang Lake, Gan River, China	Ouyang et al., 2015 [51]
*S*.*rivularis*	3	KJ434529	KJ434589	Poyang Lake, Gan River, China	Ouyang et al., 2015 [51]
*S*.*rivularis*	4	KJ434530	KJ434590	Poyang Lake, Gan River, China	Ouyang et al., 2015 [51]
*S*.*rivularis*	5	KJ434532	KJ434592	Poyang Lake, Gan River, China	Ouyang et al., 2015 [51]
*Solenaia triangularis*(Heude, 1885)	1	GQ451872	KJ434593	Gan River, China	Unpublished; Ouyang et al., 2015 [51]
*S*.*triangularis*	2	n/a	KJ434594	Poyang Lake, Gan River, China	Ouyang et al., 2015 [51]
AMBLEMINAE					
*Amblema plicata* (Say, 1817)	1	DQ648118	AY158796	USA	Elderkin et al., 2007 [105]; Serb et al., 2003 [61]
*Elliptio complanata* (Lightfoot, 1786)	1	KU906090	EF446099	USA	Unpublished; Kneeland & Rhymer, 2007 [98]
*Elliptio dilatata* (Rafinesque, 1820)	1	AF156507	DQ385872	USA	Graf & Foighil, 2000 [90]; Campbell et al., 2008 [80]
*Fusconaia askewi* (Marsh, 1896)	1	KT285626	JN180976	USA	Iii et al., 2016 [106]; Burlakova et al., 2012 [107]
*Fusconaia burkei* (Ortmann & Walker, 1922)	1	KT285627	AY158793	USA	Iii et al., 2016 [106]; Serb et al., 2003 [64]
*Fusconaia cerina* (Conrad, 1838)	1	AY613823	AY613792	USA	Campbell et al., 2005 [103]
*Pleurobema sintoxia* (Rafinesque, 1820)	1	EF033253	AY613815	USA	Chapman et al., 2008 [108]; Campbell et al., 2005 [103]
*Quadrula quadrula* (Rafinesque, 1820)	1	NC_013658	NC_013658	USA	Breton et al., 2011 [94]
*Villosa arkansasensis* (Lea, 1862)	1	KF035228	KF035359	USA	Inoue et al., 2013 [109]
OUTGROUP					
*Margaritifera dahurica* (Middendorff, 1850)		NC_023942	NC_023942	Russia	Yang et al., 2015 [110]
*Margaritifera falcata* (Gould, 1850)		NC_015476	NC_015476	USA	Breton et al., 2009 [111]

### DNA extraction, amplification and sequencing

Whole genomic DNA was extracted from preserved foot tissue using the TIANGEN TIANamp Marine Animals DNA Kit (Tiangen Biotech, Beijing, China) according to the manufacturer’s instructions. Polymerase chain reaction (PCR) primers for the two gene regions were COI (LCO1490/HCO2198) [62], and ND1 (Leu-uurF/LoGlyR) [63]. Thermal cycling conditions for both sets of primers were 98°C for 10 s, followed by 35 cycles of 94°C for 1 min, 50°C for 1 min, 72°C for 1–2 min, and a final extension of 72°C for 7 min, following the TaKaRa Ex manufacturer’s protocol. Amplified PCR products were purified and sequenced by Sangon Biotech (Shanghai). PCR product sizes for the COI and ND1 amplicons were 680 bp and 900 bp, respectively. The sequences obtained in this study have been uploaded to GenBank (Accession Numbers: MG933687-MG933805).

### DNA barcoding dataset construction

The COI and ND1 sequences of all available Chinese freshwater mussel species were downloaded from NCBI GenBank and combined with the new sequences from this study. We used DNA Collapser (http://users-birc.au.dk/biopv/php/fabox/dnacollapser.php#) to identify unique haplotypes of COI and ND1 sequences for each species, and excluded any identical sequences (Table 1). As some of the specimens used in this study were obtained from GenBank, COI or ND1 sequences are missing for some specimens. In addition, the dataset analyzed also includes DNA sequences obtained from GenBank for representatives of major clades of the Unionidae, as determined in recent studies [49, 64]. *Margaritifera falcata* and *M*. *dahurica* from the putative unionid sister family Margaritiferidae were selected as out-groups. All 57 species used in the phylogenetic analyses, including 34 Chinese species in this study, are listed in Table 1.

### Phylogenetic analyses

The COI and ND1 nucleotide sequences were translated to amino acid sequences using MEGA 5.0 [65], and aligned based on the amino acid sequences using the program MUSCLE [66] with default setting. We calculated inter- and intraspecific distances for each data set with MEGA 5.0 using the Kimura-2-parameter model [67]. Standard error was assessed using 1000 bootstrap replicates. Meanwhile, we constructed Maximum Likelihood (ML) tree based on codon position using the GTR+I+G model in MEGA5.0 for the COI and ND1 datasets separately. The results of ML analysis were shown in S1 and S2 Figs.

Using SequenceMatrix [68], the two (COI and ND1) data sets were concatenated (1011 bp) for construction of phylogenetic trees. Prior to phylogenetic analysis of the combined dataset, a partition homogeneity test was carried out in PAUP* version 4.0b10 [69] to determine if significantly different signals were being generated by the COI and ND1 fragments. The partition homogeneity test indicated there was no significant difference in signals (*P* > 0.05), and the concatenated two-loci dataset was suitable for phylogenetic construction. For the combined dataset, a single scheme with 6 partitions was applied based on genes and codon positions. The best-fit models of nucleotide substitution under the corrected Akaike Information Criterion were selected using PartitionFinder v1.1.1 [70] for each partition, of the subsequent analyses. A Bayesian topology was inferred for each dataset using MrBayes Version 2.01 [71]. The GTR+I+G model was used for the first and second COI and ND1 codon positions, while the GTR+I model was applied to third codon positions for both genes. Four chains were run simultaneously for 1 million generations and trees were sampled every 1000 generations, with a burn-in of 25%. Stationarity was considered to be reached when the average standard deviation of split frequencies was less than 0.01.

The gene and codon site-based partitioned ML analyses were performed in RAxML implemented in raxmlGUI v.1.3 [72], using the GTRGAMMAI model of nucleotide substitution with the search strategy set to rapid bootstrapping.

## Results

### Efficacy of both loci for DNA barcoding

**Fig 1:**
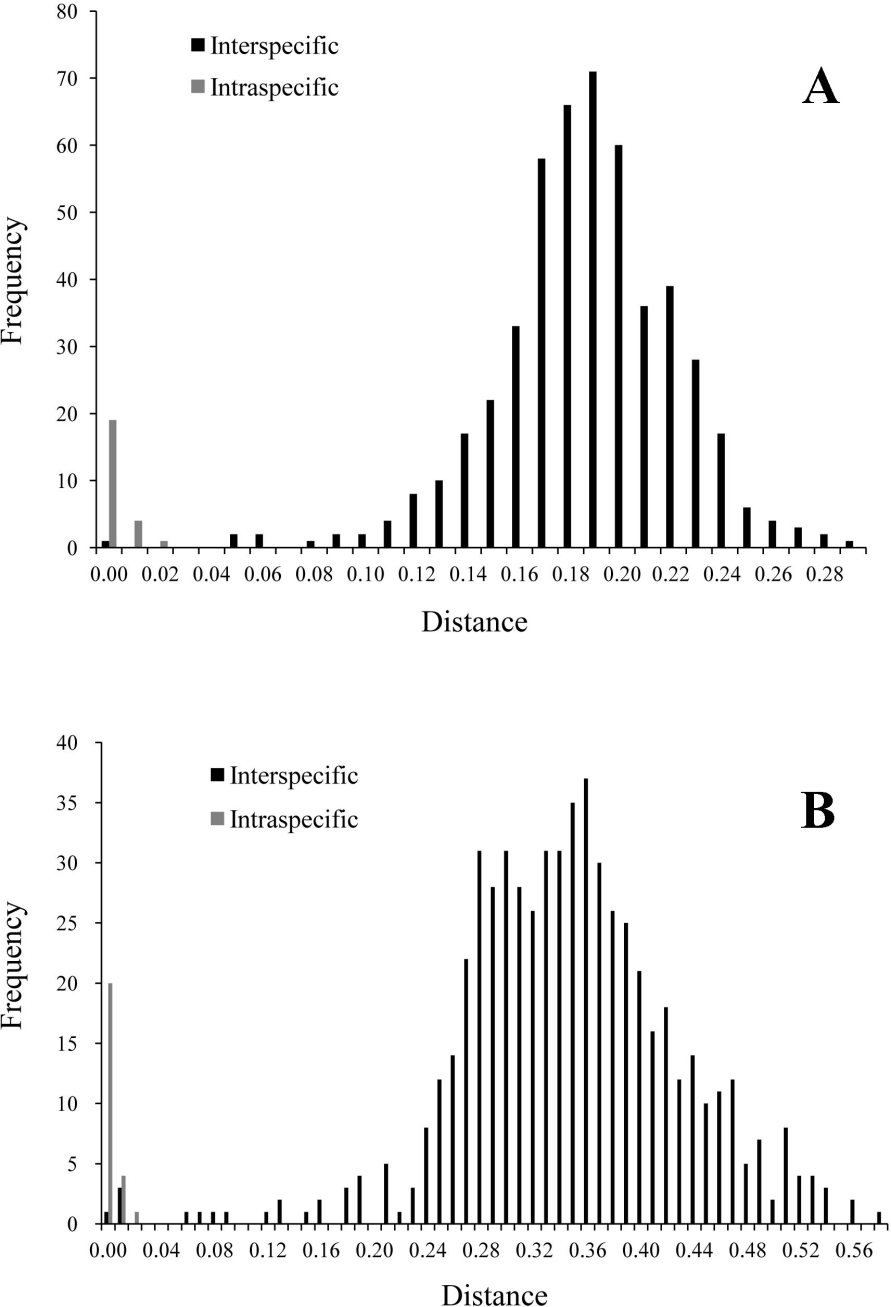
Frequency distribution of Kimura-2-parameter distances for the cytochrome oxidase c subunit I (COI) locus (A) and the nicotinamide adenine dinucleotide dehydrogenase subunit 1 (ND1) locus (B).

Initial analysis resulted in 98 COI sequences representing 32 species and 85 ND1 sequences representing 34 species in this study (Table 2). The aligned COI and ND1 sequences had a total length of 522bp and 489bp, respectively. The final alignment of ND1 sequences was trimmed to the length of the shortest sequence in the final data set. For the COI locus, average intraspecific distances calculated by the Kimura-2-parameter model ranged from 0.002–0.027 (mean = 0.007; Table 2 and Fig 1A). Interspecific genetic distances ranged from 0.05 to 0.21, except for *Anemina arcaeformis* and *A*. *globosula* with the lower interspecific genetic distance of 0.005 (see S1 Table and Fig 1A). The average interspecific distances were 10 times larger than the average intraspecific distances (Fig 1A). As a result, COI had excellent potential for species-level identification of unionids. For the ND1 locus, average intraspecific distances ranged from 0.002 to 0.024 (mean = 0.007; Table 2 and Fig 1B). The average interspecific distance of ND1 was greater than 10 times average intraspecific distance (Fig 1B). ND1 also showed excellent potential for species-level identification, as the DNA sequences exhibited larger difference between average intra- and interspecific distances than the COI gene.

**Table 2:** Mean intraspecific distances (d) and standard error (SE) assessed using 1000 bootstrap replicates based on the Kimura-2-parameter model calculated with pairwise deletion.

Species	COI			ND1		
	*n*	d	d(SE)	*n*	d	d(SE)
*Aculamprotula fibrosa*	1	NA	NA	1	NA	NA
*Aculamprotula scripta*	2	0.008	0.004	3	0.008	0.003
*Aculamprotula tientsinensis*	1	NA	NA	2	0.002	0.01
*Aculamprotula tortuosa*	6	0.011	0.003	3	0.01	0.003
*Acuticosta chinensis*	5	0.006	0.002	7	0.011	0.003
*Acuticosta ovata*	0	NA	NA	2	0.006	0.003
*Anemina angula*	2	0.004	0.003	1	NA	NA
*Anemina arcaeformis*	5	0.006	0.002	3	0.004	0.002
*Anemina globosula*	3	0.003	0.002	1	NA	NA
*Arconaia lanceolata*	1	NA	NA	3	0.011	0.004
*Cristaria plicata*	10	0.008	0.002	2	0.002	0.002
*Cuneopsis celtiformis*	3	0.004	0.002	2	0.002	0.002
*Cuneopsis heudei*	2	0.006	0.003	2	0.006	0.003
*Cuneopsis pisciculus*	4	0.008	0.003	5	0.011	0.003
*Cuneopsis rufescens*	2	0.004	0.003	1	NA	NA
*Lamprotula caveata*	4	0.004	0.002	3	0.003	0.002
*Lamprotula leaii*	3	0.009	0.003	5	0.006	0.002
*Lanceolaria grayii*	2	0.006	0.003	1	NA	NA
*Lanceolaria eucylindrica*	0	NA	NA	3	0.006	0.003
*Lanceolaria gladiola*	1	NA	NA	1	NA	NA
*Lanceolaria triformis*	3	0.005	0.003	3	0.006	0.003
*Lepidodesma languilati*	3	0.027	0.006	2	0.002	0.002
*Nodularia douglasiae*	5	0.016	0.004	1	NA	NA
*Ptychorhynchus pfisteri*	2	0.002	0.002	2	0.024	0.007
*Schistodesmus lampreyanus*	4	0.003	0.002	2	0.002	0.002
*Schistodesmus spinosus*	1	NA	NA	1	NA	NA
*Sinanodonta lucida*	1	NA	NA	2	0.002	0.002
*Sinanodonta woodiana*	4	0.004	0.002	3	0.006	0.002
*Sinanodonta elliptica*	3	0.003	0.002	4	0.006	0.002
*Sinohyriopsis cumingii*	4	0.008	0.003	3	0.006	0.003
*Solenaia carinata*	1	NA	NA	1	NA	NA
*Solenaia oleivora*	4	0.013	0.003	3	0.003	0.002
*Solenaia rivularis*	5	0.012	0.003	5	0.008	0.003
*Solenaia triangularis*	1	NA	NA	2	0.015	0.006
Mean		0.007			0.007	

n = number of individuals, COI = cytochrome oxidase c, ND1 = NADH dehydrogenase.

### Phylogenetic analyses

**Fig 2:**
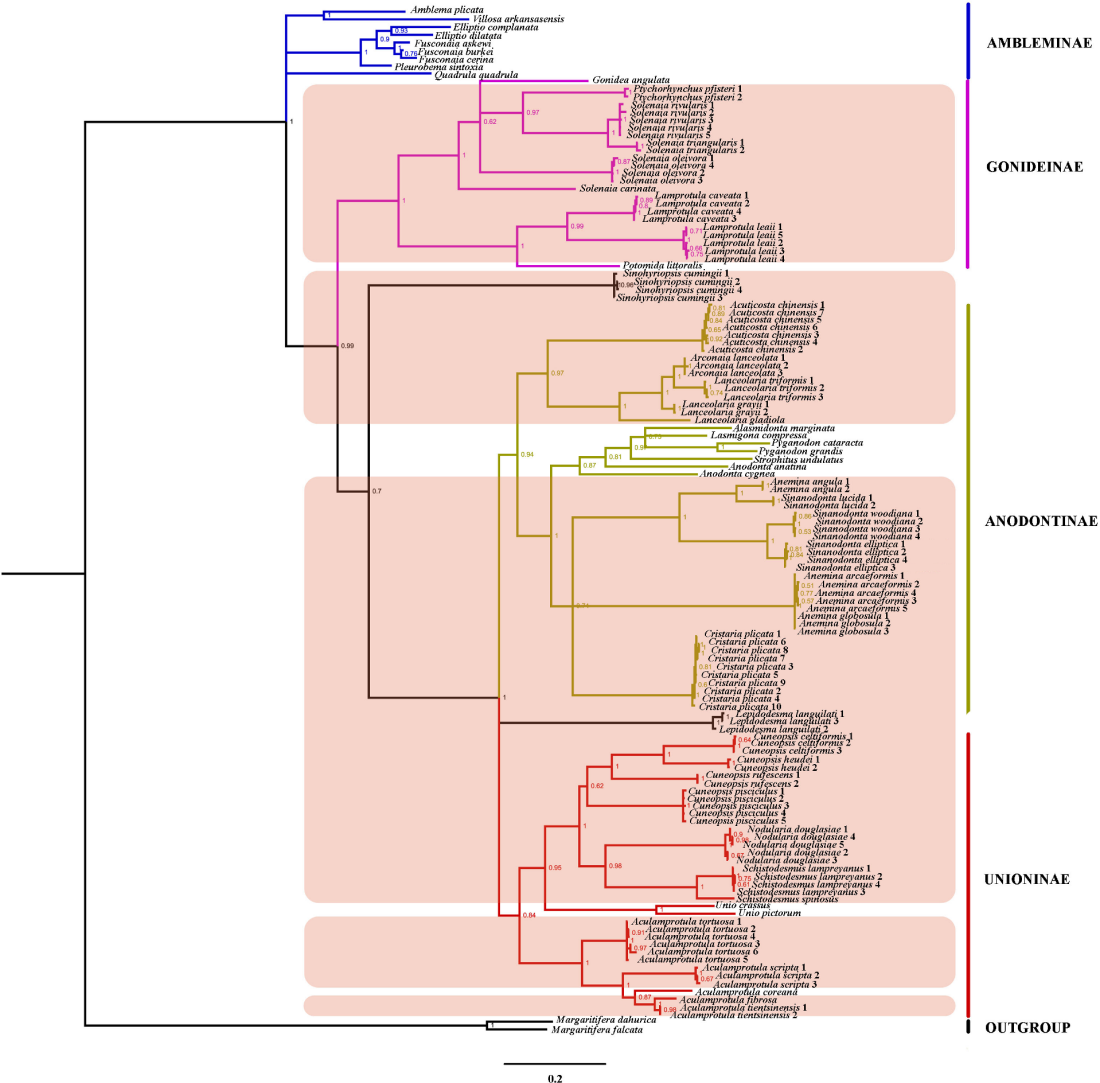
Molecular phylogeny tree of Unionidae by Bayesian Inference (BI) analyses of the DNA-barcoding (COI+ND1) dataset. The hatched section shows the Chinese mussel species in this study.

The phylogenetic trees produced by Bayesian Inference (BI) and Maximum Likelihood (ML) converged on a completely consistent topology; therefore, only the BI tree was shown here (Fig 2). Phylogenetic analyses supported the monophyly of the Unioninae, Anodontinae and Gonideninae, and the sister relationship of the Unioninae and the Anodontinae; however, the Ambleminae was recovered as a polytomy. Several Chinese genera included were supported as monophyletic groups: *Aculamprotula*, *Cuneopsis*, *Schistodesmus* and *Lamprotula*, whereas others (*Solenaia*, *Lanceolaria* and *Anemina*) were not. Based on our results, Chinese genera included in the Unioninae are: *Aculamprotula*, *Cuneopsis*, *Nodularia* and *Schistodesmus*. The Anodontinae includes the following six Chinese genera: *Cristaria*, *Arconaia*, *Acuticosta*, *Lanceolaria*, *Anemina* and *Sinoanodonta*. The Gonideninae include *Lamprotula*, *Solenaia* and *Ptychorhychus* (Fig 2). *Sinohyriopsis cumingii* was recovered as the sister taxon to the monophyletic group (Anodontinae + Unioninae) with low posterior probability, whereas *Lepidodesma languilati* was recovered as paraphyletic to the Unioninae and the Anodontinae with high posterior probability.

## Discussion

Traditionally, the taxonomic classification and identification of freshwater mussels has been mainly based on the comparative morphology of shells, especially in field surveys of freshwater mussel diversity. However, under different habitat conditions and environmental pressures the morphological characteristics of the shell, such as shell size, shape, and sculpture (e.g. ridges, bumps, and knobs) can vary significantly among populations within a species [73–74]. Using only morphological characters for unionid species identification has led to a risk of misidentification, synonymy, and a failure to describe some cryptic species [75–76]. With species as the key unit for measuring biodiversity, a failure to recognize species not only undermines biodiversity research, protection efforts and sustainable harvests and uses, but also affects biodiversity assessment efforts and the ability of resource managers to compare faunas among regions [77]. This study evaluated the utility of two mitochondrial genes widely used as DNA barcoding for 34 species of freshwater mussels in China, which not only lays a foundation for understanding the phylogenetic diversity of unionids, but also provides an important reference for assessing and comparing regional biodiversity. Furthermore, this dataset will facilitate field survey studies using eDNA and metabarcoding analyses [52] for assemblages of Chinese Unionidae in the future.

Hebert et al. [78] proposed that a 10× difference between intra- and inter-specific distances is a desirable characteristic for a barcoding locus. Both of the two loci used in this study satisfy this criterion and have excellent potential for species-level identification of Chinese unionids. The larger distance between the ranges of intra- and inter-specific distances exhibited by the ND1 gene indicates that it is more sensitive and more accurate than COI, the proposed standard barcoding locus [79], and therefore has advantages over COI for species-level identification of unionids. This is a similar result to what was found in a study on North American unionids [80]. We also found that ND1 sequence data were generally easier to obtain than COI sequence data, and that the ND1 primers had a higher rate of successful amplification, and sequence data generally were of higher quality.

After 1949, Chinese malacologists conducted a number of faunal investigations, revised classifications, and in some cases described new species [25–30]. Based on shell sculpture and hinge tooth morphology, Liu et al. [29] divided Chinese unionids into two subfamilies, the Unioninae and the Anodontinae. However, shell characteristics are not as stable as the anatomical characters (e.g. arrangement of the marsupial demibranchs in which larvae are brooded and larval morphology) and are not recommended for diagnosing higher-level taxa among freshwater mussels [81–84]. Wu et al. [32] found that larval morphology differed between members of the Unioninae and Anodontinae. For most Unioninae species, the overall shape of the glochidia was described as widely triangular. The surface of the valves of glochidia from members of the Unioninae was imperforate and included some small depressions or fovea. Whereas, for all species of Anodontinae, the overall shape of the glochidia was elongately triangular, and the surface of the larval valves included numerous perforations. On the basis of these characteristics, the genera *Arconaia*, *Lanceolaria* and *Acuticosta* were placed into the Unioninae [29, 32], which has been supported by later molecular studies [48, 50–51]. Recently, however, Lopes-Lima et al. [49] placed the genera *Arconaia* and *Lanceolaria* into the Anodontinae based on phylogenetic analysis of COI and 28S rRNA sequences. Our study supports not only changing the subfamilial affinities of *Arconaia* and *Lanceolaria* to the Anodontinae, but also indicates with high confidence that the genus *Acuticosta* also belongs in the Anodontinae. Renewed examination of the anatomy of these genera is warranted to determine if characteristics that support the molecular classification presented here and in other publications can be identified.

The taxonomy of Chinese anodontines had been controversial for a long time. Chinese freshwater malacologists consistently used the generic name *Anodonta* in the taxonomy, ecology and molecular biology, and considered all Chinese anodontines as monophyletic. But, other research [9, 85] indicated that the genus *Anodonta sensu stricto* was restricted to Western North America and Western Europe ranging as far east as Lake Baikal, and that Chinese anodontines were instead members of the genera *Anemina* and *Sinoanodonta*. Our study provides additional molecular evidence indicating that Chinese *Anodonta sensu lato* are polyphyletic, and supports dividing Chinese *Anodonta* into the genera *Anemina* and *Sinoanodonta*. (See and Figs 2, S1 and S2). *Anemina angula* is a Chinese endemic species, and it was placed in *Anemina* by Prozorova et al. [86] and Graf and Cummings [3] on the basis of morphological characters. Our results instead indicate it has closer affinities to *Sinoanodonta*.

The combined COI and ND1 dataset indicates that the Ambleminae is the sister group to the rest of the Unionidae. The phylogeny of Lopes-Lima et al. [49] indicated that the 4 subfamilies were divided into 2 branches: (Anodontinae + Unioninae) and (Ambleminae + Gonideninae). Bolotov et al. [64] thought the placement of the Pseudodontinae as a tribe within Gonideinae was incorrect and proposed that they actually represent a separate subfamily. But in general, his phylogenetic analyses supported the Unionidae phylogenetic framework established by Lopes-Lima et al. [49]. The subfamily-level phylogenetic relationships in our study differ from the above-mentioned results. In addition, our analyses were unable to place the genera *Sinohyriopsis* and *Lepidodesma* in the phylogeny with confidence. Some researchers have placed these genera into the Gonideninae and the Unioninae, respectively, based on the analysis of complete mitochondrial genomes [45, 47].

For the purpose of recognizing and delimiting species in this study we are employing the monophyly version of the phylogenetic species concept [87–89]. In our DNA barcoding dataset for 34 freshwater mussel species, the interspecific genetic distance between *Anemina arcaeformis* and *A*. *globosula* (COI: d = 0.006; ND1: d = 0.006) were much smaller than those of other species (See S1 and S2 Tables). At the same time, they clustered with each other in an evolutionary lineage and formed a monophyletic group (S1 and S2 Tables and Fig 2). According to the phylogenetic species concept, our results do not support recognizing *A*. *arcaeformis* and *A*. *globosula* as distinct species. Data from nuclear gene sequences and morphological data are needed to corroborate the findings based on this mtDNA dataset.

DNA barcoding and modern molecular phylogenetic analyses of the Unionidae have had significant impacts on our understanding of the biogeography of this important family of freshwater bivalves. The pattern being revealed is one that includes both highly endemic subfamilies (e.g. Ambleminae in North and Central America) and some subfamilies that are found on several continents (e.g. Anodontinae and Gonideinae) [3–4, 49]. China, and in particular the Yangtze Basin is recognized a region of high unionid diversity which includes a number of endemic taxa [7]. The majority of unionid taxa in China and the surrounding region have never been included in a molecular phylogenetic analysis [49]. In view of the generally imperiled conservation status for freshwater mussels in China [7, 11–12], it is of crucial importance to develop a phylogenetic framework for Chinese taxa to assist with species delineation and determining priorities for species conservation. This study provides an improved foundation for the systematics and taxonomy of Unionidae in China and serves as a reference for future studies of Chinese freshwater mussel diversity.

## Supporting information

S1 Table Interspecific distances of 32 putative species using COI loci. The lower left is the interspecific genetic distance; the upper right is the standard error.
(DOCX)

S2 Table Interspecific distances of 34 putative species using ND1 loci. The lower left is the interspecific genetic distance; the upper right is the standard error.
(DOCX)

S1 Fig ML tree of Chinese freshwater mussels inferred from the first subunit of the cytochrome oxidase c (COI) gene. (TIF)

S2 Fig ML tree of Chinese freshwater mussels inferred from the first subunit of the NADH dehydrogenase (ND1) gene. (TIF)

## References

[1] Vaughn CC, Spooner DE. Unionid mussels influence macroinvertebrate assemblage structure in streams. Freshwater Science. 2006; 25: 691–700.

[2] Vaughn CC, Nichols SJ, Spooner DE. Community and foodweb ecology of freshwater mussels. Journal of the North American Benthological Society. 2008; 27: 409–423.

[3] Graf DL, Cummings KS. Review of the systematics and global diversity of freshwater mussel species (Bivalvia: Unionoida). Journal of Molluscan Studies. 2007; 73: 291–314.

[4] Bogan AE. Global diversity of freshwater mussels (Mollusca, Bivalvia) in freshwater. Hydrobiologia. 2008; 595: 139–147.

[5] IUCN. The IUCN Red List of Threatened Species. Version 2015–4. 2015.

[6] Lydeard C, Cowie RH, Ponder WF, Bogan AE, Bouchet P, Clark SA, et al The global decline of nonmarine mollusks. BioScience. 2004; 54: 321–330.

[7] Zieritz A, Bogan AE, Froufe E, Klishko O, Kondo T, Kovitvadhi U, et al Diversity, biogeography and conservation of freshwater mussels (Bivalvia: Unionida) in East and Southeast Asia. Hydrobiologia. 2018 10.1007/s10750-017-3104-8

[8] He J, Zhuang Z. The Freshwater Bivalves of China. Harxheim, Germany: Conchbooks; 2013.

[9] Shu FY, Wang HJ, Cui YD, Wang HZ. Diversity and distribution pattern of freshwater molluscs in the Yangtze River basin. Acta Hydrobiologica Sinica. 2014; 38: 19–26.

[10] Wu XP, Liang YL, Wang HZ, Xie ZC, Ouyang S. Distribution and species diversity of freshwater mollusca of lakes along mid-lower reaches of the Yangtze River. Journal of Lake Sciences. 2000; 12: 111–118.

[11] Shu FY, Wang HJ, Pan BZ, Liu XQ, Wang HZ. Assessment of species status of mollusca in the mid-lower Yangtze Lakes. Acta Hydrobiologica Sinica. 2009; 33: 1051–1058.

[12] Wu RW, Gao BY, Lan ZC, Zhang MH, Ouyang S, Wu XP. Predicting local colonization and extinction rates of freshwater mussels based on biological traits in a case of Lake Poyang. Journal of Lake Sciences. 2017; 29: 678–686.

[13] Heude RP. Conchyliologie fluviatile de la province de Nanking, 1 Paris: Librairie F. Savy; 1875.

[14] Heude RP. Conchyliologie fluviatile de la province de Nanking et la Chine centrale. 2 Paies: Librairie F. Scvy; 1877a.

[15] Heude RP. Conchyliologie fluviatile de la province de Nanking et la Chine centrale. 3 Paies: Librairie F. Scvy; 1877b.

[16] Heude RP. Conchyliologie fluviatile de la province de Nanking et la Chine centrale. 4 Paies: Librairie F. Scvy; 1878.

[17] Heude RP. Conchyliologie fluviatile de la province de Nanking et la Chine centrale. 5 Paies: Librairie F. Scvy; 1879.

[18] Heude RP. Conchyliologie fluviatile de la province de Nanking et la Chine centrale. 6 Paies: Librairie F. Scvy; 1880a.

[19] Heude RP. Conchyliologie fluviatile de la province de Nanking et la Chine centrale. 10 Paies: Librairie F. Scvy; 1880b.

[20] Heude RP. Conchyliologie fluviatile de la province de Nanking et la Chine centrale. 7 Paies: Librairie F. Scvy; 1881.

[21] Heude RP. Conchyliologie fluviatile de la province de Nanking et la Chine centrale. 8 Paies: Librairie F. Scvy; 1883.

[22] Heude RP. Conchyliologie fluviatile de la province de Nanking et la Chine centrale. 9 Paies: Librairie F. Scvy; 1885.

[23] Simpson CT. New and Unfigured Unionidae. Proceedings of the Academy of Natural Sciences of Philadelphia. 1900; 52: 74–86.

[24] Haas F. Superfamilia Unionacea. Berlin: Walter de Gruyter; 1969.

[25] Lin ZT. Unionidae (Mollusca) of Poyang Lake, Kiangsi Province, China. Acta Zoologica Sinica. 1962; 14: 249–260.

[26] Tchang S, Li SC. Bivalves (Mollusca) of the Poyang Lake and surrounding waters, Kiangsi Province, China, with description of a new species. Acta Zoologica Sinica. 1965; 17: 309–319.

[27] Tchang S, Li SC, Liu YY. Bivalves (Mollusca) of Tung-Ting Lake and its surrounding waters, Hunan Province, China. Acta Zoologica Sinica. 1965; 17: 197–216.

[28] Liu YY, Huang YY. Notes on freshwater mollusks of the San-Men-Hsia Reservoir on the Yellow River and its neighboring regions, China. Acta Zoologica Sinica. 1964; 16: 429–439.

[29] Liu YY, Zhang WZ, Wang QX, Wang EY. Economic Fauna of China-Freshwater Mollusk. Beijing: Science Press; 1979.

[30] Liu YY, Zhang WZ, Wang YX. Bivalves (Mollusca) of the Tai Hu Lake and its surrounding waters, Jiangsu Province, China. Acta Zoologica Sinica. 1980; 26: 365–369.

[31] Wei QS, Fu CH. Comparative studies on morphology of the glochidia of six mussel species (Mollusca: Unionidae). Acta Hydrobiologica Sinica. 1994; 18: 303–308.

[32] Wu XP, Liang Y, Wang H, Ouyang S. Morphological characters of glochidia of Unionidae and the taxonomic significance. Acta Hydrobiologica Sinica. 1999; 23: 139–147.

[33] Wu XP, Liang YL, Wang HZ. A comparative study on glochidial morphology of Unionidae (Bivalvia)-I. *Unio douglasiae*, *Cuneopsis pisciulus*, *Acuticosta chinensis* and *Acuticosta ovata*. Acta Hydrobiologica Sinica. 1999; 23: 141–145.

[34] Wu XP, Liang YL, Wang HZ, Ouyang S. A comparative study on glochidial morphology of unionidae (Bivalvia) ii. *Lanceolaria*, *Lamprotula*, *Hyriopsis* and *Cristaria*. Acta Hydrobiologica Sinica. 2000; 24: 252–256.

[35] Shu FY, Ouyang S, Wu XP. Glochidial morphology of two species of the genus *Schistodesmus* (Bivalvia: Unionidae) from Lake Poyang, China. American Malacological Bulletin. 2012; 30: 329–333.

[36] Hebert PDN, Gregory TR. The promise of DNA barcoding for taxonomy. Systematic Biology. 2005; 54: 852–859. 10.1080/10635150500354886 16243770

[37] Liu ZJ, Cordes JF. DNA marker technologies and their applications in aquaculture genetics. Biotechnology Bulletin. 2004; 238: 1–37.

[38] Huang XC, Rong J, Liu Y, Zhang MH, Wan Y, Ouyang S, et al The complete maternally and paternally inherited mitochondrial genomes of the endangered freshwater mussel *Solenaia carinatus* (Bivalvia: Unionidae) and implications for Unionidae taxonomy. Plos One. 2013; 8: e84352 10.1371/journal.pone.0084352 24358356 PMC3866145

[39] Huang XC, Zhou CH, Ouyang S, Wu XP. The complete F-type mitochondrial genome of threatened Chinese freshwater mussel *Solenaia oleivora* (Bivalvia: Unionidae: Gonideinae). Mitochondrial DNA. 2015; 26: 263–264. 10.3109/19401736.2013.823190 24021015

[40] Song XL, Ouyang S, Zhou CH, Wu XP. Complete maternal mitochondrial genome of freshwater mussel *Anodonta lucida* (Bivalvia: Unionidae: Anodontinae). Mitochondrial DNA Part A. 2016; 27: 549–550. 10.3109/19401736.2014.905852 24708121

[41] Pfeiffer JM, Graf DL. Re-analysis confirms the polyphyly of *Lamprotula* Simpson, 1900 (Bivalvia: Unionidae). Journal of Molluscan Studies. 2013; 79: 249–256.

[42] Wang GL, Gao XR, Li JL. Complete F-type mitochondrial genome of Chinese freshwater mussel *Lamprotula tortuosa*. Mitochondrial DNA. 2013; 24: 513–515. 10.3109/19401736.2013.770508 23521580

[43] Wang Q, Ma L, Li L, Feng RJ, Mu H, Wang X, et al The complete maternal mitochondrial genome sequence of *Cuneopsis heudei* (Bivalvia: Unionoida: Unionidae). Conservation Genetics Resources. 2017 10.1007/s12686-017-0876-0

[44] Wu RW, An CT, Wu XP, Zhou CH, Ouyang S. Complete maternal mitochondrial genome of freshwater mussel *Aculamprotula tientsinensis* (Bivalvia: Unionidae: Unioninae). Mitochondrial DNA Part A. 2016; 27: 4520–4521. 10.3109/19401736.2015.1101543 26540022

[45] Wu RW, Wang S, Liu YT, Liu XJ, Zhou CH, Ouyang S, et al Characterization and phylogenetic analysis of the complete maternal mitochondrial genome of freshwater mussel *Aculamprotula scripta* (Bivalvia: Unionidae: Unioninae). Conservation Genetics Resources. 2017 Available from: 10.1007/s12686-017-0914-y

[46] Zhou CH, Huang XC, Ouyang S, Ouyang JX, Wu XP. Characterization of the complete maternal mitochondrial genome of *Ptychorhynchus pfisteri* (Bivalvia: Unionidae: Gonideinae). Conservation Genetics Resources. 2016; 9: 233–235.

[47] Zhou CH, Ouyang S, Wu XP, Ding MH. The complete maternal mitochondrial genome of rare Chinese freshwater mussel *Lepidodesma languilati* (Bivalvia: Unionidae: Unioninae). Mitochondrial DNA Part A. 2016; 27: 4615–4616. 10.3109/19401736.2015.1101583 26678883

[48] Zhou CH., Ouyang S, Wu XP, Li M. Phylogeny of the genus *Lamprotula* (Unionidae) in China based on mitochondrial DNA sequences of 16S rRNA and ND1 genes. Acta Zoologica Sinica. 2007; 53: 1024–1030.

[49] Lopes-Lima M, Froufe E, Ghamizi M, Mock K, Kebapci Ü, Klishko O, et al Phylogeny of the most species rich freshwater bivalve family (Bivalvia: Unionida: Unionidae): Defining modern subfamilies and tribes. Molecular Phylogenetics & Evolution. 2017; 106: 174–191. 27621130 10.1016/j.ympev.2016.08.021

[50] Huang YY, Ouyang S, Wu XP, Liu H. Phylogeny of the Unionidae based on partial mitochondrial 16S rRNA sequences. Acta Hydrobiologica Sinica. 2003; 27: 258–263.

[51] Ouyang JX, Su JH, Ouyang S, Zhou CH, Wu XP, Li SB. DNA barcoding and molecular phylogenetic analysis of freshwater Unionids. Journal of Agricultural Biotechnology. 2015; 23: 779–787.

[52] Baird DJ, Sweeney BW. Applying DNA barcoding in benthology: the state of the science. Journal of the North American Benthological Society. 2011; 30: 122–124.

[53] Boyer SL, Howe AA, Juergens NW, Hove M. A DNA-barcoding approach to identifying juvenile freshwater mussels (Bivalvia:Unionidae) recovered from naturally infested fishes. Journal of the North American Benthological Society. 2011; 30: 182–194.

[54] Kneeland SC, Rhymer JM. Determination of fish host use by wild populations of rare freshwater mussels using a molecular identification key to identify glochidia. Freshwater Science. 2015; 27: 150–160.

[55] Mikkelsen NT, Schander C, Willassen E. Local scale DNA barcoding of bivalves (Mollusca): a case study. Zoologica Scripta. 2007; 36: 455–463.

[56] Roe KJ. Phylogenetic Analysis of the Freshwater Mussel Genus *Ptychobranchus* (Bivalvia: Unionidae). American Malacological Bulletin. 2013; 31: 257–265.

[57] Roe KJ, Lydeard C. Molecular systematics of the freshwater mussel genus *Potamilus* (Bivalvia: Unionidae). Malacologia. 1998; 39: 195–205.

[58] Zieritz A, Yasaeng P, Razak NFA, Hongtrakul V, Kovitvadhi U, Kanchanaketu. Development and evaluation of hotshot protocols for cost- and time- effective extraction of PCR-ready DNA from single freshwater mussel larvae (Bivalvia: Unionida). Journal of Molluscan Studies. 2018; 84: 198–201.

[59] Cai RX. Fauma of Zhejiang: Mollusks. Zhejiang Science and Technology Publish House 1991.

[60] Graf DL, Cummings KS. The MUSSEL Project Database. 2018. http://www.mussel-project.net/

[61] Wang YN, Zhang GR, Wei KJ, Gardner JPA. Reproductive traits of the threatened freshwater mussel *Solenaia oleivora* (Bivalvia: Unionidae) from the middle Yangtze River. Journal of Molluscan Studies. 2015; 81: 23–29.

[62] Folmer O, Black M, Hoeh W, Lutz R, Vrijenhoek R. DNA primers for amplification of mitochondrial cytochrome c oxidase subunit I from diverse metazoan invertebrates. Molecular Marine Biology & Biotechnology. 1994; 3: 294–299. 7881515

[63] Serb JM, Buhay JE, Lydeard C. Molecular systematics of the North American freshwater bivalve genus *Quadrula* (Unionidae: Ambleminae) based on mitochondrial ND1 sequences. Molecular Phylogenetics & Evolution. 2003; 28 10.1016/S1055-7903(03)00026-5 12801467

[64] Bolotov IN, Kondakov AV, Vikhrev IV, Aksenova OV, Bespalaya YV, Gofarov MY, et al Ancient river inference explains exceptional oriental freshwater mussel radiations. Scientific Reports. 2017; 7: 2135 10.1038/s41598-017-02312-z 28522869 PMC5437074

[65] Tamura K, Peterson D, Peterson N, Stecher G, Nei M, Kumar S. Mega5: Molecular evolutionary genetics analysis using maximum likelihood, evolutionary distance, and maximum parsimony methods. Molecular Biology & Evolution. 2011; 28: 2731–2739. 21546353 10.1093/molbev/msr121 PMC3203626

[66] Edgar RC. Muscle: multiple sequence alignment with high accuracy and high throughput. Nucleic Acids Research. 2004; 32: 1792–1797. 10.1093/nar/gkh340 15034147 PMC390337

[67] Kimura M. A simple method for estimating evolutionary rates of base substitutions through comparative studies of nucleotide sequences. Journal of Molecular Evolution. 1980; 16: 111–120. 7463489 10.1007/BF01731581

[68] Vaidya G, Lohman DJ, Meier R. SequenceMatrix: concatenation software for the fast assembly of multi-gene datasets with character set and codon information. Cladistics. 2011; 27: 171–180. 10.1111/j.1096-0031.2010.00329.x 34875773

[69] Swofford DL. PAUP*. Phylogenetic Analysis Using Parsimony (*and Other Methods). Version 4.0b10. Mccarthy; 2003. doi: 10.1111/j.0014-3820.2002.tb00191.x.

[70] Lanfear R, Calcott B, Ho SY, Guindon S. Partitionfinder: combined selection of partitioning schemes and substitution models for phylogenetic analyses. Molecular Biology & Evolution. 2012; 29: 1695–1701. 22319168 10.1093/molbev/mss020

[71] Ronquist F, Teslenko M, Mark PV, Ayres DL, Darling A, Hohna S, et al Mrbayes 3.2: Efficient Bayesian phylogenetic inference and model choice across a large model space. Systematic Biology. 2012; 61: 539–542. 10.1093/sysbio/sys029 22357727 PMC3329765

[72] Stamatakis A. RAxML version 8: a tool for phylogenetic analysis and post-analysis of large phylogenies. Bioinformatics. 2014; 30: 1312–1313. 10.1093/bioinformatics/btu033 24451623 PMC3998144

[73] Zieritz A, Hoffman JI, Amos W, Aldridge DC. Phenotypic plasticity and genetic isolation-by-distance in the freshwater mussel *Unio pictorum* (Mollusca: Unionoida). Evolutionary Ecology. 2010; 24: 923–938.

[74] Zieritz A, Sartori AF, Bogan AE, Aldridge DC. Reconstructing the evolution of umbonal sculptures in the Unionida. Journal of Zoological Systematics & Evolutionary Research, 2015; 53: 76–86.

[75] Klishko OK, Lopes-lima M, Froufe E, Bogan AE, Abakumova VY. Unravelling the systematics of *Nodularia* (Bivalvia, Unionidae) species from eastern Russia. Systematics and Biodiversity. 2017 10.1080/14772000.2017.1383527

[76] Klishko OK, Lopes-Lima M, Froufe E, Bogan AE. Are *Cristaria herculea* (Middendorff, 1847) and *Cristaria plicata* (Leach, 1815) (Bivalvia, Unionidae) separate species? Zookeys. 2014; 438: 1–15. 10.3897/zookeys.438.7493 PMC4155721 25197215

[77] Hong DY. Biodiversity pursuits need a scientific and operative species concept. Biodiversity Science. 2016; 24: 979–999.

[78] Hebert PDN, Stoeckle MY, Zemlak TS, Francis CM. Identification of birds through DNA barcodes. Plos Biology. 2004; 2: e312 10.1371/journal.pbio.0020312 15455034 PMC518999

[79] Hebert PDN, Cywinska A, Ball SL, Dewaard JR. Biological identifications through DNA barcodes. Proceedings of the Royal Society B: Biological Sciences. 2003; 270: 313–321. 10.1098/rspb.2002.2218 12614582 PMC1691236

[80] Campbell DC, Johnson PD, Williams JD, Rindsberg AK, Serb JM, Small KK, et al Identification of 'extinct' freshwater mussel species using DNA barcoding. Molecular Ecology Resources. 2008; 8: 711–724. 10.1111/j.1755-0998.2008.02108.x 21585879

[81] Ortmann AE. Notes upon the families and genera of the Najades. Annals of the Carnegie Museum. 1912; 8: 222–365.

[82] Heard WH. Anatomical systematics of freshwater mussels. Malacological Review. 1974; 7: 41–42.

[83] Heard WH, Guckert RH. A re-evaluation of the Recent Unionacea (Pelecypoda) of North America. Malacologia. 1971; 10: 333–355.

[84] Davis GM, Fuller SLH. Genetic relationships among Recent Unionacea (Bivalvia) of North America. Malacologia. 1981; 20: 217–253.

[85] Klishko OK, Lopeslima M, Bogan AE, Matafonov DV, Froufe E. Morphological and molecular analyses of Anodontinae species (Bivalvia, Unionidae) of Lake Baikal and Transbaikalia. 2018; Plos One: 13: e0194944 10.1371/journal.pone.0194944 29630628 PMC5890983

[86] Prozorova LA, Sayenko EM, Bogatov VV, Wu M. Bivalves of the Yangtze River drainage. The Bulletin of the Russian Far East Malacological Society. 2005; 9: 46–58.

[87] Donoghue MJ. A critique of the biological species concept and recommendations for a phylogenetic alternative. Bryologist. 1985; 88: 172–181.

[88] De QK. Species concepts and species delimitation. Systematic Biology. 2007; 56: 879–886. 10.1080/10635150701701083 18027281

[89] Wiens JJ. Species delimitation: new approaches for discovering diversity. Systematic Biology. 2007; 56: 875–878. 10.1080/10635150701748506 18027280

[90] Graf DL, Ó’Foighil D. The evolution of brooding characters among the freshwater pearly mussels (Bivalvia: Unioniodea) of North America. Journal of Molluscan Studies. 2000; 66: 157–170.

[91] Soroka M. Complete female mitochondrial genome of *Anodonta anatina* (Mollusca: Unionidae): confirmation of a novel protein-coding gene (F ORF). Mitochondrial DNA. 2015; 26: 267–269. 10.3109/19401736.2013.823176 24020999

[92] Soroka M, Burzyński A. Hermaphroditic freshwater mussel *Anodonta cygnea* does not have supranumerary open reading frames in the mitogenome. Mitochondrial DNA, 2017; 2: 862–864. 10.1080/23802359.2017.1407705 PMC7800200 33474013

[93] Wang G, Chen M, Li J. Complete F-type mitochondrial genome of freshwater mussel *Lanceolaria glayana*. Mitochondrial DNA. 2016; 27: 846–847. 10.3109/19401736.2014.919470 24865898

[94] Breton S, Stewart DT, Shepardson S, Trdan RJ, Bogan AE, Chapman EG, et al Novel protein genes in animal mtDNA: a new sex determination system in freshwater mussels (Bivalvia: Unionoida)? Molecular Biology & Evolution. 2011; 28: 1645–1659. 21172831 10.1093/molbev/msq345 PMC3107663

[95] Krebs RA, Allen BD, Evans NM, Zanatta DT. Mitochondrial DNA structure of *Pyganodon grandis* (Bivalvia: Unionidae) from the Lake Erie watershed and selected locations in its northern distribution. American Malacological Bulletin. 2015; 33: 1–9.

[96] Smith CH, Johnson NA, Pfeiffer JM, Gangloff MM. Molecular and morphological data reveal paraphyly and speciation in imperiled freshwater mussels (*Anodontoides* and *Strophitus*). Molecular Phylogenetics & Evolution. 2017 10.1016/j.ympev.2017.10.018 29074460

[97] Hoeh WR, Mcalpine DF, Hebda A, Stewart DT. mtDNA and AFLP markers demonstrate limited genetic differentiation within the *Pyganodon cataracta*–*Pyganodon fragilis* freshwater mussel complex in Atlantic Canada. Canadian Journal of Zoology. 2012; 90: 1307–1319.

[98] Kneeland SC, Rhymer JM. A molecular identification key for freshwater mussel glochidia encysted on naturally parasitized fish hosts in Maine, USA. Journal of Molluscan Studies. 2007; 73: 279–282.

[99] Bogan AE, Hoeh WR. On becoming cemented: evolutionary relationships among the genera in the freshwater bivalve family Etheriidae (Bivalvia: Unionoida). Geological Society London Special Publications. 2000; 77: 159–168.

[100] Soroka M. Characteristics of mitochondrial DNA of unionid bivalves (Mollusca: Bivalvia: Unionidae). I. Detection and characteristics of doubly uniparental inheritance (DUI) of unionid mitochondrial DNA. Géotechnique. 2010; 18: 147–188.

[101] Soroka M, Burzyński A. Complete sequences of maternally inherited mitochondrial genomes in mussels *Unio pictorum* (Bivalvia, Unionidae). Journal of Applied Genetics. 2010; 51: 469–476. 10.1007/BF03208876 21063064

[102] Gustafson RG, Iwamoto EM. A DNA-based identification key to Pacific Northwest freshwater mussel glochidia: importance to salmonid and mussel conservation. Northwest Science. 2005; 79: 233–245.

[103] Campbell DC, Serb JM, Buhay JE, Roe KJ, Minton RL, Lydeard C. Phylogeny of North American amblemines (Bivalvia, Unionoida): prodigious polyphyly proves pervasive across genera. Invertebrate Biology. 2005; 124: 131–164.

[104] Froufe E, Gan HM, Lee YP, Carneiro J, Varandas S, Teixeira A, et al The male and female complete mitochondrial genome sequences of the endangered freshwater mussel *Potomida littoralis* (Cuvier, 1798) (Bivalvia: Unionidae). Mitochondrial DNA Part A. 2016; 27: 3571–3572. 10.3109/19401736.2015.1074223 27158872

[105] Elderkin CL, Christian AD, Vaughn CC, Metcalfesmith JL, Berg DJ. Population genetics of the freshwater mussel *Amblema plicata* (say 1817) (Bivalvia: Unionidae): evidence of high dispersal and post-glacial colonization. Conservation Genetics. 2007; 8: 355–372.

[106] Pfeiffer JM, Johnson NA, Randklev CR, Howells RG, Williams JD. Generic reclassification and species boundaries in the rediscovered freshwater mussel *‘ Quadrula’ mitchelli* (Simpson in Dall, 1896). Conservation Genetics. 2016; 17: 279–292.

[107] Burlakova LE, Campbell D, Karatayev AY, Barclay D. Distribution, genetic analysis and conservation priorities for rare Texas freshwater molluscs in the genera *Fusconaia* and *Pleurobema* (Bivalvia: Unionidae). Aquatic Biosystems. 2012; 8: 1–15. 10.1186/2046-9063-8-1 22731520 PMC3422191

[108] Chapman EG, Piontkivska H, Walker JM, Stewart DT, Curole JP, Hoeh WR. Extreme primary and secondary protein structure variability in the chimeric male-transmitted cytochrome c oxidase subunit ii protein in freshwater mussels: evidence for an elevated amino acid substitution rate in the face of domain-specific purifying selection. BMC Evolutionary Biology. 2008; 8: 165 Available from: 10.1186/1471-2148-8-165 18513440 PMC2430956

[109] Inoue K, Hayes DM, Harris JL, Christian AD. Phylogenetic and morphometric analyses reveal ecophenotypic plasticity in freshwater mussels *Obovaria jacksoniana* and *Villosa arkansasensis* (Bivalvia: Unionidae). Ecology & Evolution. 2013; 3: 2670–2683. 24567831 10.1002/ece3.649 PMC3930048

[110] Yang S, Mi Z, Tao G, Liu X, Wei M, Wang H. The complete mitochondrial genome sequence of *Margaritiana dahurica* Middendorff. Mitochondrial DNA. 2015; 26: 716–717. 10.3109/19401736.2013.845755 24617476

[111] Breton S, Beaupré HD, Stewart DT, Piontkivska H, Karmakar M, Bogan AE, et al Comparative mitochondrial genomics of freshwater mussels (Bivalvia: Unionoida) with doubly uniparental inheritance of mtDNA: gender-specific open reading frames and putative origins of replication. Genetics. 2009; 183: 1575–1589. 10.1534/genetics.109.110700 19822725 PMC2787441

