# Supplementary material for: Testing the utility of DNA barcodes and a preliminary phylogenetic framework for Chinese freshwater mussels (Bivalvia: Unionidae) from the middle and lower Yangtze River
Source: PLoS One. 2018 Aug 8;13(8):e0200956. doi: 10.1371/journal.pone.0200956 (PMC6082535; doi:10.1371/journal.pone.0200956)
Supplement: S1 Table — The lower left is the interspecific genetic distance; the upper right is the standard error. (DOCX) [file pone.0200956.s001.docx]

Supplementary Table S1 Interspecific distances of 32 Chinese Unionidae species using COI loci. The lower left is the interspecific genetic distance, the upper right is the standard error.

| *Aculamprotula fibrosa* |  | 0.019 | 0.013 | 0.020 | 0.029 | 0.029 | 0.035 | 0.035 | 0.026 | 0.029 | 0.027 | 0.026 | 0.027 | 0.027 | 0.026 | 0.030 | 0.026 | 0.029 | 0.025 | 0.024 | 0.026 | 0.030 | 0.024 | 0.025 | 0.031 | 0.027 | 0.026 | 0.027 | 0.031 | 0.030 | 0.026 | 0.030 |
| --- | --- | --- | --- | --- | --- | --- | --- | --- | --- | --- | --- | --- | --- | --- | --- | --- | --- | --- | --- | --- | --- | --- | --- | --- | --- | --- | --- | --- | --- | --- | --- | --- |
| *A. scripta* | 0.119 |  | 0.018 | 0.023 | 0.026 | 0.025 | 0.034 | 0.034 | 0.027 | 0.027 | 0.024 | 0.024 | 0.022 | 0.025 | 0.024 | 0.031 | 0.024 | 0.026 | 0.025 | 0.024 | 0.024 | 0.026 | 0.025 | 0.025 | 0.029 | 0.024 | 0.026 | 0.029 | 0.028 | 0.031 | 0.027 | 0.031 |
| *A.tientsinensis* | 0.067 | 0.109 |  | 0.017 | 0.026 | 0.028 | 0.030 | 0.030 | 0.027 | 0.031 | 0.024 | 0.024 | 0.027 | 0.026 | 0.026 | 0.031 | 0.022 | 0.026 | 0.025 | 0.027 | 0.026 | 0.028 | 0.024 | 0.024 | 0.029 | 0.026 | 0.027 | 0.029 | 0.031 | 0.031 | 0.028 | 0.031 |
| *A.tortuosa* | 0.128 | 0.153 | 0.108 |  | 0.021 | 0.028 | 0.025 | 0.025 | 0.025 | 0.028 | 0.023 | 0.024 | 0.026 | 0.026 | 0.023 | 0.029 | 0.019 | 0.027 | 0.022 | 0.024 | 0.023 | 0.027 | 0.021 | 0.022 | 0.026 | 0.024 | 0.023 | 0.026 | 0.028 | 0.028 | 0.025 | 0.027 |
| *Acuticosta chinensis* | 0.215 | 0.199 | 0.192 | 0.163 |  | 0.025 | 0.026 | 0.026 | 0.021 | 0.029 | 0.023 | 0.021 | 0.023 | 0.022 | 0.025 | 0.024 | 0.021 | 0.019 | 0.023 | 0.024 | 0.022 | 0.027 | 0.024 | 0.023 | 0.023 | 0.026 | 0.024 | 0.024 | 0.024 | 0.024 | 0.027 | 0.023 |
| *Anemina angula* | 0.218 | 0.201 | 0.215 | 0.213 | 0.190 |  | 0.030 | 0.030 | 0.025 | 0.028 | 0.029 | 0.025 | 0.026 | 0.024 | 0.025 | 0.027 | 0.026 | 0.021 | 0.023 | 0.026 | 0.025 | 0.028 | 0.023 | 0.024 | 0.015 | 0.021 | 0.023 | 0.026 | 0.031 | 0.026 | 0.025 | 0.029 |
| *Anemina arcaeformis* | 0.281 | 0.270 | 0.236 | 0.197 | 0.191 | 0.232 |  | 0.002 | 0.030 | 0.040 | 0.026 | 0.029 | 0.031 | 0.025 | 0.027 | 0.029 | 0.028 | 0.027 | 0.029 | 0.030 | 0.028 | 0.030 | 0.033 | 0.030 | 0.031 | 0.025 | 0.030 | 0.029 | 0.029 | 0.028 | 0.031 | 0.030 |
| *A.globosula* | 0.278 | 0.265 | 0.232 | 0.194 | 0.188 | 0.229 | 0.005 |  | 0.030 | 0.039 | 0.026 | 0.028 | 0.031 | 0.025 | 0.027 | 0.029 | 0.028 | 0.027 | 0.029 | 0.030 | 0.028 | 0.030 | 0.033 | 0.030 | 0.031 | 0.025 | 0.030 | 0.029 | 0.029 | 0.028 | 0.030 | 0.030 |
| *Arconaia lanceolata* | 0.188 | 0.209 | 0.197 | 0.178 | 0.156 | 0.185 | 0.220 | 0.219 |  | 0.026 | 0.024 | 0.024 | 0.020 | 0.021 | 0.025 | 0.024 | 0.020 | 0.015 | 0.011 | 0.024 | 0.023 | 0.026 | 0.023 | 0.023 | 0.026 | 0.025 | 0.025 | 0.025 | 0.023 | 0.022 | 0.025 | 0.025 |
| *Cristaria plicata* | 0.232 | 0.202 | 0.241 | 0.215 | 0.219 | 0.225 | 0.291 | 0.285 | 0.187 |  | 0.029 | 0.029 | 0.026 | 0.030 | 0.029 | 0.030 | 0.023 | 0.028 | 0.027 | 0.029 | 0.027 | 0.028 | 0.023 | 0.025 | 0.032 | 0.030 | 0.030 | 0.032 | 0.032 | 0.031 | 0.028 | 0.030 |
| *Cuneopsis celtiformis* | 0.201 | 0.169 | 0.174 | 0.166 | 0.171 | 0.222 | 0.194 | 0.194 | 0.182 | 0.223 |  | 0.018 | 0.019 | 0.022 | 0.023 | 0.026 | 0.026 | 0.026 | 0.023 | 0.026 | 0.019 | 0.026 | 0.024 | 0.023 | 0.028 | 0.026 | 0.029 | 0.023 | 0.026 | 0.025 | 0.026 | 0.028 |
| *C.heudei* | 0.182 | 0.175 | 0.167 | 0.182 | 0.159 | 0.194 | 0.220 | 0.215 | 0.177 | 0.222 | 0.116 |  | 0.020 | 0.019 | 0.022 | 0.026 | 0.023 | 0.023 | 0.019 | 0.025 | 0.021 | 0.025 | 0.024 | 0.020 | 0.026 | 0.025 | 0.026 | 0.022 | 0.024 | 0.025 | 0.024 | 0.026 |
| *C.pisciculus* | 0.210 | 0.170 | 0.194 | 0.205 | 0.179 | 0.213 | 0.244 | 0.241 | 0.159 | 0.202 | 0.134 | 0.138 |  | 0.019 | 0.026 | 0.025 | 0.027 | 0.022 | 0.020 | 0.024 | 0.019 | 0.027 | 0.023 | 0.021 | 0.027 | 0.027 | 0.026 | 0.024 | 0.027 | 0.027 | 0.027 | 0.026 |
| *C.rufescens* | 0.188 | 0.188 | 0.186 | 0.186 | 0.162 | 0.184 | 0.190 | 0.187 | 0.149 | 0.243 | 0.148 | 0.131 | 0.136 |  | 0.023 | 0.023 | 0.023 | 0.020 | 0.021 | 0.024 | 0.020 | 0.025 | 0.022 | 0.019 | 0.023 | 0.024 | 0.024 | 0.023 | 0.028 | 0.026 | 0.026 | 0.025 |
| *Lamprotula caveata* | 0.205 | 0.192 | 0.202 | 0.181 | 0.188 | 0.194 | 0.207 | 0.204 | 0.198 | 0.225 | 0.177 | 0.172 | 0.197 | 0.177 |  | 0.018 | 0.024 | 0.027 | 0.024 | 0.027 | 0.024 | 0.020 | 0.024 | 0.023 | 0.026 | 0.026 | 0.026 | 0.020 | 0.021 | 0.021 | 0.019 | 0.022 |
| *L.leaii* | 0.242 | 0.247 | 0.247 | 0.236 | 0.179 | 0.217 | 0.228 | 0.225 | 0.188 | 0.242 | 0.209 | 0.204 | 0.202 | 0.179 | 0.121 |  | 0.025 | 0.025 | 0.025 | 0.025 | 0.026 | 0.024 | 0.025 | 0.026 | 0.029 | 0.028 | 0.029 | 0.022 | 0.023 | 0.023 | 0.023 | 0.023 |
| *Lanceolaria gladiola* | 0.189 | 0.165 | 0.166 | 0.128 | 0.150 | 0.183 | 0.202 | 0.200 | 0.121 | 0.165 | 0.182 | 0.169 | 0.198 | 0.164 | 0.178 | 0.195 |  | 0.019 | 0.020 | 0.022 | 0.023 | 0.024 | 0.021 | 0.022 | 0.028 | 0.026 | 0.025 | 0.026 | 0.026 | 0.026 | 0.024 | 0.022 |
| *L.grayii* | 0.204 | 0.195 | 0.194 | 0.198 | 0.140 | 0.157 | 0.198 | 0.195 | 0.091 | 0.204 | 0.184 | 0.163 | 0.157 | 0.135 | 0.212 | 0.190 | 0.117 |  | 0.015 | 0.024 | 0.023 | 0.023 | 0.025 | 0.022 | 0.022 | 0.025 | 0.024 | 0.025 | 0.025 | 0.023 | 0.025 | 0.023 |
| *L.triformis* | 0.185 | 0.185 | 0.184 | 0.168 | 0.166 | 0.166 | 0.212 | 0.210 | 0.056 | 0.197 | 0.170 | 0.138 | 0.152 | 0.153 | 0.187 | 0.191 | 0.125 | 0.091 |  | 0.022 | 0.024 | 0.024 | 0.024 | 0.022 | 0.024 | 0.022 | 0.025 | 0.026 | 0.023 | 0.022 | 0.024 | 0.027 |
| *Lepidodesma languilati* | 0.174 | 0.170 | 0.193 | 0.178 | 0.182 | 0.198 | 0.232 | 0.231 | 0.182 | 0.219 | 0.203 | 0.191 | 0.188 | 0.185 | 0.217 | 0.200 | 0.158 | 0.180 | 0.165 |  | 0.022 | 0.024 | 0.023 | 0.020 | 0.027 | 0.027 | 0.027 | 0.023 | 0.026 | 0.026 | 0.027 | 0.031 |
| *Nodularia douglasiae* | 0.206 | 0.187 | 0.197 | 0.179 | 0.168 | 0.192 | 0.225 | 0.222 | 0.177 | 0.206 | 0.132 | 0.152 | 0.142 | 0.142 | 0.184 | 0.210 | 0.167 | 0.170 | 0.182 | 0.182 |  | 0.026 | 0.021 | 0.018 | 0.024 | 0.025 | 0.023 | 0.023 | 0.028 | 0.026 | 0.027 | 0.026 |
| *Ptychorhynchus pfisteri* | 0.241 | 0.214 | 0.224 | 0.221 | 0.204 | 0.222 | 0.237 | 0.236 | 0.203 | 0.233 | 0.209 | 0.200 | 0.211 | 0.198 | 0.146 | 0.168 | 0.187 | 0.175 | 0.186 | 0.189 | 0.210 |  | 0.026 | 0.025 | 0.030 | 0.026 | 0.026 | 0.025 | 0.021 | 0.022 | 0.017 | 0.022 |
| *Schistodesmus lampreyanus* | 0.176 | 0.192 | 0.177 | 0.151 | 0.181 | 0.174 | 0.252 | 0.251 | 0.172 | 0.180 | 0.171 | 0.171 | 0.158 | 0.149 | 0.193 | 0.203 | 0.147 | 0.177 | 0.174 | 0.179 | 0.148 | 0.208 |  | 0.012 | 0.023 | 0.025 | 0.025 | 0.025 | 0.026 | 0.026 | 0.022 | 0.026 |
| *S.spinosus* | 0.177 | 0.178 | 0.174 | 0.159 | 0.171 | 0.177 | 0.233 | 0.230 | 0.169 | 0.190 | 0.161 | 0.137 | 0.140 | 0.126 | 0.181 | 0.203 | 0.152 | 0.153 | 0.163 | 0.148 | 0.126 | 0.192 | 0.061 |  | 0.023 | 0.024 | 0.023 | 0.023 | 0.030 | 0.024 | 0.024 | 0.028 |
| *Sinanodonta lucida* | 0.237 | 0.227 | 0.226 | 0.204 | 0.171 | 0.087 | 0.232 | 0.234 | 0.189 | 0.248 | 0.203 | 0.199 | 0.221 | 0.170 | 0.205 | 0.227 | 0.208 | 0.160 | 0.179 | 0.215 | 0.183 | 0.238 | 0.170 | 0.181 |  | 0.022 | 0.023 | 0.027 | 0.035 | 0.026 | 0.030 | 0.030 |
| *S.woodiana* | 0.204 | 0.182 | 0.190 | 0.181 | 0.194 | 0.144 | 0.193 | 0.191 | 0.183 | 0.222 | 0.194 | 0.189 | 0.197 | 0.179 | 0.193 | 0.207 | 0.191 | 0.182 | 0.167 | 0.198 | 0.185 | 0.202 | 0.179 | 0.168 | 0.150 |  | 0.010 | 0.026 | 0.031 | 0.029 | 0.029 | 0.028 |
| *S. elliptica* | 0.195 | 0.194 | 0.203 | 0.176 | 0.183 | 0.172 | 0.227 | 0.225 | 0.183 | 0.215 | 0.214 | 0.204 | 0.196 | 0.175 | 0.198 | 0.217 | 0.184 | 0.179 | 0.184 | 0.200 | 0.180 | 0.209 | 0.186 | 0.166 | 0.163 | 0.051 |  | 0.028 | 0.033 | 0.028 | 0.029 | 0.025 |
| *Sinohyriopsis cumingii* | 0.226 | 0.223 | 0.228 | 0.210 | 0.180 | 0.199 | 0.224 | 0.222 | 0.195 | 0.249 | 0.176 | 0.171 | 0.181 | 0.178 | 0.154 | 0.167 | 0.202 | 0.192 | 0.198 | 0.185 | 0.190 | 0.184 | 0.197 | 0.182 | 0.209 | 0.191 | 0.209 |  | 0.021 | 0.021 | 0.019 | 0.021 |
| *Solenaia carinata* | 0.252 | 0.217 | 0.238 | 0.218 | 0.183 | 0.241 | 0.232 | 0.229 | 0.174 | 0.257 | 0.206 | 0.194 | 0.205 | 0.222 | 0.149 | 0.170 | 0.193 | 0.191 | 0.173 | 0.209 | 0.224 | 0.141 | 0.196 | 0.224 | 0.271 | 0.239 | 0.260 | 0.151 |  | 0.020 | 0.020 | 0.023 |
| *S.oleivora* | 0.243 | 0.253 | 0.244 | 0.235 | 0.198 | 0.204 | 0.226 | 0.224 | 0.175 | 0.249 | 0.204 | 0.200 | 0.221 | 0.217 | 0.151 | 0.171 | 0.205 | 0.180 | 0.172 | 0.215 | 0.228 | 0.161 | 0.209 | 0.196 | 0.208 | 0.233 | 0.232 | 0.162 | 0.145 |  | 0.020 | 0.022 |
| *S.rivularis* | 0.208 | 0.213 | 0.222 | 0.202 | 0.217 | 0.196 | 0.237 | 0.236 | 0.194 | 0.218 | 0.195 | 0.181 | 0.205 | 0.203 | 0.144 | 0.171 | 0.188 | 0.195 | 0.183 | 0.206 | 0.213 | 0.114 | 0.163 | 0.181 | 0.239 | 0.222 | 0.218 | 0.134 | 0.138 | 0.147 |  | 0.018 |
| *S.triangularis* | 0.260 | 0.250 | 0.261 | 0.227 | 0.181 | 0.236 | 0.241 | 0.239 | 0.195 | 0.228 | 0.213 | 0.205 | 0.203 | 0.192 | 0.167 | 0.174 | 0.170 | 0.176 | 0.205 | 0.248 | 0.208 | 0.153 | 0.192 | 0.211 | 0.230 | 0.213 | 0.197 | 0.153 | 0.166 | 0.168 | 0.123 |  |
