# Supplementary material for: Testing the utility of DNA barcodes and a preliminary phylogenetic framework for Chinese freshwater mussels (Bivalvia: Unionidae) from the middle and lower Yangtze River
Source: PLoS One. 2018 Aug 8;13(8):e0200956. doi: 10.1371/journal.pone.0200956 (PMC6082535; doi:10.1371/journal.pone.0200956)
Supplement: S2 Table — The lower left is the interspecific genetic distance; the upper right is the standard error. (DOCX) [file pone.0200956.s002.docx]

Supplementary Table S2 Interspecific distances of 34 Chinese Unionidae species using ND1 loci. The lower left is the interspecific genetic distance, the upper right is the standard error.

| *Aculamprotula fibrosa* |  | 0.026 | 0.007 | 0.025 | 0.035 | 0.036 | 0.042 | 0.042 | 0.042 | 0.041 | 0.035 | 0.025 | 0.031 | 0.037 | 0.037 | 0.040 | 0.045 | 0.037 | 0.042 | 0.037 | 0.046 | 0.037 | 0.040 | 0.049 | 0.035 | 0.042 | 0.044 | 0.050 | 0.048 | 0.050 | 0.040 | 0.046 | 0.050 | 0.050 |
| --- | --- | --- | --- | --- | --- | --- | --- | --- | --- | --- | --- | --- | --- | --- | --- | --- | --- | --- | --- | --- | --- | --- | --- | --- | --- | --- | --- | --- | --- | --- | --- | --- | --- | --- |
| *A.scripta* | 0.177 |  | 0.025 | 0.031 | 0.034 | 0.036 | 0.038 | 0.047 | 0.046 | 0.044 | 0.034 | 0.036 | 0.038 | 0.042 | 0.046 | 0.042 | 0.038 | 0.038 | 0.039 | 0.041 | 0.046 | 0.047 | 0.038 | 0.047 | 0.041 | 0.046 | 0.038 | 0.046 | 0.048 | 0.051 | 0.037 | 0.047 | 0.051 | 0.050 |
| *A.tientsinensis* | 0.064 | 0.165 |  | 0.024 | 0.035 | 0.036 | 0.041 | 0.039 | 0.039 | 0.037 | 0.036 | 0.026 | 0.030 | 0.035 | 0.036 | 0.039 | 0.045 | 0.035 | 0.039 | 0.035 | 0.042 | 0.037 | 0.037 | 0.049 | 0.034 | 0.039 | 0.044 | 0.051 | 0.046 | 0.049 | 0.038 | 0.044 | 0.048 | 0.048 |
| *A.tortuosa* | 0.172 | 0.212 | 0.160 |  | 0.028 | 0.030 | 0.043 | 0.041 | 0.042 | 0.042 | 0.033 | 0.035 | 0.036 | 0.033 | 0.036 | 0.042 | 0.045 | 0.040 | 0.039 | 0.040 | 0.041 | 0.039 | 0.036 | 0.054 | 0.039 | 0.041 | 0.039 | 0.039 | 0.037 | 0.049 | 0.043 | 0.050 | 0.043 | 0.042 |
| *Acuticosta chinensis* | 0.276 | 0.266 | 0.271 | 0.216 |  | 0.004 | 0.043 | 0.036 | 0.038 | 0.040 | 0.033 | 0.037 | 0.037 | 0.032 | 0.037 | 0.037 | 0.040 | 0.038 | 0.043 | 0.038 | 0.040 | 0.038 | 0.033 | 0.046 | 0.040 | 0.046 | 0.039 | 0.041 | 0.037 | 0.048 | 0.040 | 0.039 | 0.054 | 0.053 |
| *A.ovara* | 0.279 | 0.278 | 0.278 | 0.230 | 0.013 |  | 0.044 | 0.036 | 0.038 | 0.040 | 0.034 | 0.037 | 0.039 | 0.033 | 0.037 | 0.039 | 0.041 | 0.039 | 0.044 | 0.037 | 0.040 | 0.040 | 0.034 | 0.048 | 0.042 | 0.047 | 0.039 | 0.044 | 0.038 | 0.048 | 0.042 | 0.041 | 0.055 | 0.055 |
| *Anemina angula* | 0.332 | 0.284 | 0.321 | 0.331 | 0.343 | 0.345 |  | 0.042 | 0.042 | 0.043 | 0.038 | 0.045 | 0.044 | 0.043 | 0.044 | 0.048 | 0.056 | 0.036 | 0.046 | 0.045 | 0.051 | 0.049 | 0.039 | 0.044 | 0.042 | 0.045 | 0.016 | 0.031 | 0.027 | 0.066 | 0.047 | 0.051 | 0.051 | 0.051 |
| *A.arcaeformis* | 0.332 | 0.366 | 0.306 | 0.314 | 0.295 | 0.289 | 0.332 |  | 0.003 | 0.039 | 0.039 | 0.042 | 0.041 | 0.039 | 0.046 | 0.051 | 0.048 | 0.039 | 0.048 | 0.038 | 0.045 | 0.048 | 0.036 | 0.046 | 0.048 | 0.051 | 0.040 | 0.042 | 0.038 | 0.065 | 0.049 | 0.053 | 0.072 | 0.071 |
| *A.globosula* | 0.328 | 0.360 | 0.303 | 0.320 | 0.303 | 0.301 | 0.329 | 0.006 |  | 0.038 | 0.041 | 0.043 | 0.043 | 0.040 | 0.047 | 0.053 | 0.049 | 0.040 | 0.046 | 0.038 | 0.047 | 0.048 | 0.037 | 0.044 | 0.049 | 0.052 | 0.042 | 0.043 | 0.039 | 0.063 | 0.048 | 0.051 | 0.070 | 0.069 |
| *Arconaia lanceolata* | 0.309 | 0.310 | 0.283 | 0.295 | 0.287 | 0.285 | 0.349 | 0.310 | 0.300 |  | 0.035 | 0.039 | 0.039 | 0.036 | 0.046 | 0.045 | 0.054 | 0.020 | 0.028 | 0.005 | 0.013 | 0.037 | 0.041 | 0.053 | 0.050 | 0.046 | 0.048 | 0.048 | 0.041 | 0.052 | 0.040 | 0.044 | 0.043 | 0.043 |
| *Cristaria plicata* | 0.265 | 0.248 | 0.264 | 0.241 | 0.262 | 0.268 | 0.295 | 0.307 | 0.313 | 0.283 |  | 0.030 | 0.038 | 0.032 | 0.036 | 0.039 | 0.042 | 0.036 | 0.044 | 0.036 | 0.040 | 0.035 | 0.038 | 0.043 | 0.034 | 0.036 | 0.037 | 0.046 | 0.036 | 0.049 | 0.044 | 0.041 | 0.049 | 0.048 |
| *Cuneopsis celtiformis* | 0.188 | 0.274 | 0.187 | 0.256 | 0.287 | 0.291 | 0.377 | 0.360 | 0.368 | 0.306 | 0.245 |  | 0.028 | 0.032 | 0.032 | 0.041 | 0.044 | 0.033 | 0.042 | 0.036 | 0.043 | 0.041 | 0.037 | 0.049 | 0.033 | 0.035 | 0.042 | 0.045 | 0.043 | 0.053 | 0.040 | 0.042 | 0.051 | 0.050 |
| *C.heudei* | 0.221 | 0.280 | 0.216 | 0.278 | 0.302 | 0.317 | 0.356 | 0.332 | 0.339 | 0.305 | 0.298 | 0.187 |  | 0.032 | 0.039 | 0.046 | 0.046 | 0.035 | 0.040 | 0.037 | 0.045 | 0.043 | 0.037 | 0.041 | 0.038 | 0.039 | 0.041 | 0.044 | 0.041 | 0.046 | 0.044 | 0.046 | 0.053 | 0.051 |
| *C.pisciculus* | 0.265 | 0.300 | 0.254 | 0.237 | 0.250 | 0.252 | 0.329 | 0.317 | 0.317 | 0.277 | 0.259 | 0.230 | 0.240 |  | 0.035 | 0.041 | 0.042 | 0.034 | 0.039 | 0.036 | 0.040 | 0.037 | 0.033 | 0.046 | 0.039 | 0.044 | 0.039 | 0.046 | 0.038 | 0.055 | 0.044 | 0.045 | 0.046 | 0.046 |
| *C.rufescens* | 0.279 | 0.329 | 0.265 | 0.258 | 0.284 | 0.290 | 0.340 | 0.352 | 0.360 | 0.348 | 0.279 | 0.240 | 0.277 | 0.260 |  | 0.046 | 0.044 | 0.037 | 0.035 | 0.041 | 0.048 | 0.038 | 0.035 | 0.049 | 0.033 | 0.037 | 0.038 | 0.044 | 0.037 | 0.054 | 0.041 | 0.048 | 0.051 | 0.051 |
| *Lamprotula caveata* | 0.327 | 0.365 | 0.321 | 0.342 | 0.317 | 0.328 | 0.409 | 0.419 | 0.425 | 0.365 | 0.334 | 0.342 | 0.393 | 0.346 | 0.377 |  | 0.034 | 0.044 | 0.048 | 0.045 | 0.045 | 0.043 | 0.043 | 0.047 | 0.049 | 0.044 | 0.052 | 0.044 | 0.043 | 0.051 | 0.042 | 0.046 | 0.039 | 0.039 |
| *L.leaii* | 0.359 | 0.313 | 0.349 | 0.366 | 0.329 | 0.338 | 0.453 | 0.390 | 0.393 | 0.409 | 0.338 | 0.365 | 0.376 | 0.345 | 0.354 | 0.242 |  | 0.052 | 0.052 | 0.052 | 0.052 | 0.046 | 0.047 | 0.049 | 0.040 | 0.050 | 0.048 | 0.045 | 0.048 | 0.046 | 0.040 | 0.043 | 0.048 | 0.047 |
| *Lanceolaria eucylindrica* | 0.292 | 0.284 | 0.277 | 0.293 | 0.292 | 0.293 | 0.298 | 0.328 | 0.325 | 0.133 | 0.283 | 0.270 | 0.279 | 0.268 | 0.297 | 0.356 | 0.417 |  | 0.027 | 0.020 | 0.023 | 0.037 | 0.036 | 0.045 | 0.046 | 0.040 | 0.041 | 0.054 | 0.044 | 0.050 | 0.041 | 0.045 | 0.045 | 0.046 |
| *L.gladiola* | 0.311 | 0.278 | 0.286 | 0.281 | 0.324 | 0.329 | 0.345 | 0.376 | 0.365 | 0.193 | 0.322 | 0.333 | 0.316 | 0.302 | 0.268 | 0.387 | 0.406 | 0.187 |  | 0.028 | 0.031 | 0.041 | 0.043 | 0.050 | 0.041 | 0.047 | 0.050 | 0.045 | 0.043 | 0.061 | 0.039 | 0.051 | 0.047 | 0.047 |
| *L.grayii* | 0.284 | 0.291 | 0.265 | 0.277 | 0.277 | 0.270 | 0.348 | 0.307 | 0.304 | 0.018 | 0.279 | 0.285 | 0.286 | 0.276 | 0.321 | 0.353 | 0.394 | 0.133 | 0.191 |  | 0.013 | 0.038 | 0.042 | 0.053 | 0.048 | 0.047 | 0.047 | 0.045 | 0.040 | 0.052 | 0.037 | 0.045 | 0.045 | 0.046 |
| *L.triformis* | 0.335 | 0.329 | 0.314 | 0.282 | 0.286 | 0.284 | 0.393 | 0.360 | 0.367 | 0.070 | 0.309 | 0.331 | 0.350 | 0.303 | 0.351 | 0.360 | 0.404 | 0.153 | 0.210 | 0.065 |  | 0.043 | 0.047 | 0.066 | 0.052 | 0.045 | 0.050 | 0.054 | 0.049 | 0.052 | 0.043 | 0.046 | 0.046 | 0.046 |
| *Lepidodesma languilati* | 0.282 | 0.354 | 0.285 | 0.302 | 0.297 | 0.309 | 0.403 | 0.380 | 0.377 | 0.297 | 0.281 | 0.322 | 0.321 | 0.281 | 0.307 | 0.345 | 0.359 | 0.308 | 0.318 | 0.295 | 0.346 |  | 0.042 | 0.059 | 0.044 | 0.047 | 0.055 | 0.055 | 0.048 | 0.055 | 0.041 | 0.049 | 0.052 | 0.052 |
| *Nodularia douglasiae* | 0.301 | 0.288 | 0.277 | 0.268 | 0.253 | 0.257 | 0.304 | 0.288 | 0.295 | 0.318 | 0.281 | 0.295 | 0.277 | 0.250 | 0.263 | 0.359 | 0.370 | 0.304 | 0.334 | 0.322 | 0.365 | 0.330 |  | 0.045 | 0.036 | 0.036 | 0.039 | 0.043 | 0.038 | 0.049 | 0.050 | 0.040 | 0.041 | 0.041 |
| *Ptychorhynchus pfisteri* | 0.395 | 0.393 | 0.388 | 0.410 | 0.385 | 0.400 | 0.375 | 0.381 | 0.369 | 0.441 | 0.368 | 0.391 | 0.351 | 0.369 | 0.410 | 0.370 | 0.390 | 0.379 | 0.422 | 0.434 | 0.517 | 0.467 | 0.368 |  | 0.070 | 0.067 | 0.049 | 0.048 | 0.044 | 0.055 | 0.043 | 0.038 | 0.040 | 0.040 |
| *Schistodesmus lampreyanus* | 0.267 | 0.316 | 0.253 | 0.285 | 0.309 | 0.323 | 0.339 | 0.366 | 0.374 | 0.387 | 0.263 | 0.256 | 0.279 | 0.288 | 0.248 | 0.403 | 0.337 | 0.357 | 0.307 | 0.369 | 0.388 | 0.328 | 0.265 | 0.539 |  | 0.019 | 0.041 | 0.047 | 0.045 | 0.052 | 0.047 | 0.049 | 0.057 | 0.057 |
| *S.spinosus* | 0.304 | 0.330 | 0.284 | 0.298 | 0.342 | 0.347 | 0.345 | 0.386 | 0.394 | 0.351 | 0.290 | 0.274 | 0.282 | 0.309 | 0.277 | 0.361 | 0.387 | 0.315 | 0.353 | 0.355 | 0.338 | 0.338 | 0.254 | 0.525 | 0.121 |  | 0.046 | 0.050 | 0.046 | 0.048 | 0.047 | 0.046 | 0.052 | 0.051 |
| *Sinanodonta lucida* | 0.351 | 0.290 | 0.340 | 0.312 | 0.319 | 0.314 | 0.099 | 0.330 | 0.343 | 0.388 | 0.289 | 0.355 | 0.335 | 0.310 | 0.315 | 0.439 | 0.393 | 0.335 | 0.376 | 0.379 | 0.397 | 0.434 | 0.303 | 0.434 | 0.332 | 0.349 |  | 0.035 | 0.031 | 0.063 | 0.053 | 0.052 | 0.049 | 0.049 |
| *S.woodiana* | 0.386 | 0.354 | 0.385 | 0.307 | 0.337 | 0.349 | 0.217 | 0.332 | 0.337 | 0.378 | 0.341 | 0.375 | 0.358 | 0.374 | 0.350 | 0.376 | 0.377 | 0.426 | 0.362 | 0.357 | 0.416 | 0.426 | 0.328 | 0.402 | 0.372 | 0.384 | 0.249 |  | 0.014 | 0.062 | 0.052 | 0.048 | 0.055 | 0.054 |
| *S. elliptica* | 0.367 | 0.358 | 0.355 | 0.292 | 0.294 | 0.306 | 0.190 | 0.296 | 0.300 | 0.327 | 0.269 | 0.360 | 0.340 | 0.312 | 0.290 | 0.375 | 0.396 | 0.351 | 0.345 | 0.321 | 0.379 | 0.377 | 0.293 | 0.384 | 0.357 | 0.359 | 0.223 | 0.080 |  | 0.059 | 0.044 | 0.048 | 0.051 | 0.050 |
| *Sinohyriopsis cumingii* | 0.392 | 0.386 | 0.391 | 0.376 | 0.389 | 0.391 | 0.494 | 0.475 | 0.464 | 0.420 | 0.382 | 0.419 | 0.384 | 0.408 | 0.439 | 0.409 | 0.380 | 0.402 | 0.472 | 0.416 | 0.408 | 0.458 | 0.387 | 0.427 | 0.405 | 0.375 | 0.480 | 0.468 | 0.456 |  | 0.048 | 0.053 | 0.049 | 0.048 |
| *Solenaia carinata* | 0.332 | 0.318 | 0.316 | 0.344 | 0.329 | 0.335 | 0.395 | 0.393 | 0.387 | 0.339 | 0.363 | 0.346 | 0.364 | 0.368 | 0.348 | 0.337 | 0.315 | 0.348 | 0.335 | 0.319 | 0.350 | 0.331 | 0.396 | 0.330 | 0.364 | 0.363 | 0.442 | 0.419 | 0.356 | 0.399 |  | 0.038 | 0.038 | 0.038 |
| *S.oleivora* | 0.373 | 0.390 | 0.356 | 0.403 | 0.311 | 0.324 | 0.425 | 0.449 | 0.439 | 0.364 | 0.333 | 0.354 | 0.355 | 0.367 | 0.409 | 0.375 | 0.328 | 0.372 | 0.411 | 0.369 | 0.369 | 0.395 | 0.339 | 0.300 | 0.415 | 0.374 | 0.441 | 0.402 | 0.403 | 0.422 | 0.288 |  | 0.038 | 0.037 |
| *S.rivularis* | 0.401 | 0.393 | 0.381 | 0.345 | 0.423 | 0.431 | 0.429 | 0.534 | 0.521 | 0.351 | 0.395 | 0.405 | 0.438 | 0.383 | 0.411 | 0.314 | 0.371 | 0.357 | 0.381 | 0.362 | 0.365 | 0.421 | 0.352 | 0.302 | 0.456 | 0.426 | 0.414 | 0.444 | 0.404 | 0.385 | 0.302 | 0.293 |  | 0.003 |
| *S.triangularis* | 0.397 | 0.386 | 0.379 | 0.340 | 0.419 | 0.429 | 0.424 | 0.530 | 0.518 | 0.350 | 0.390 | 0.403 | 0.423 | 0.379 | 0.406 | 0.312 | 0.365 | 0.358 | 0.381 | 0.361 | 0.366 | 0.421 | 0.348 | 0.299 | 0.454 | 0.418 | 0.411 | 0.434 | 0.397 | 0.375 | 0.298 | 0.288 | 0.010 |  |
