# Supplementary figures and images for: Testing the utility of DNA barcodes and a preliminary phylogenetic framework for Chinese freshwater mussels (Bivalvia: Unionidae) from the middle and lower Yangtze River
Source: PLoS One. 2018 Aug 8;13(8):e0200956. doi: 10.1371/journal.pone.0200956 (PMC6082535; doi:10.1371/journal.pone.0200956)

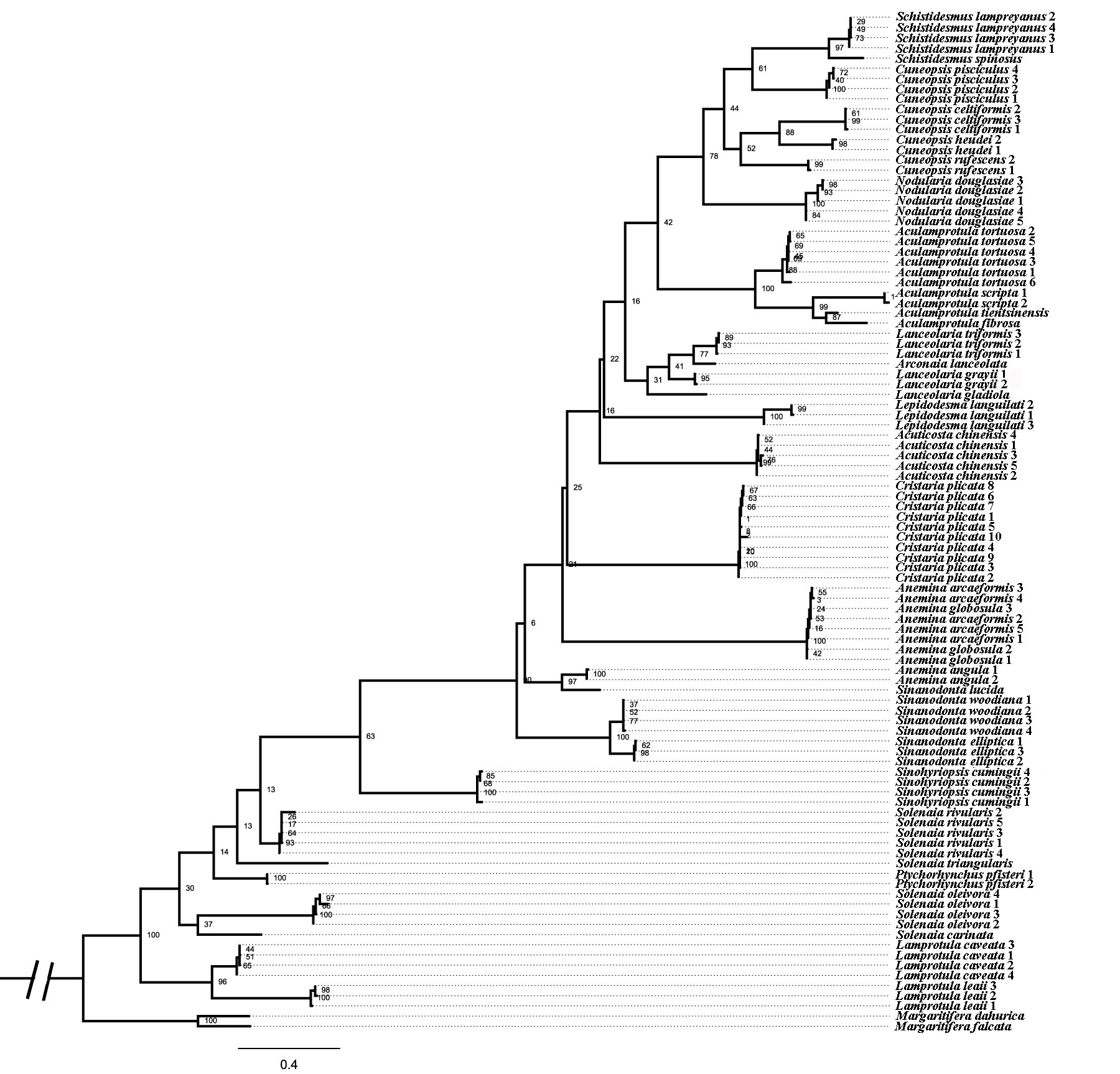

Supplement: S1 Fig — (TIF) [file pone.0200956.s003.tif]

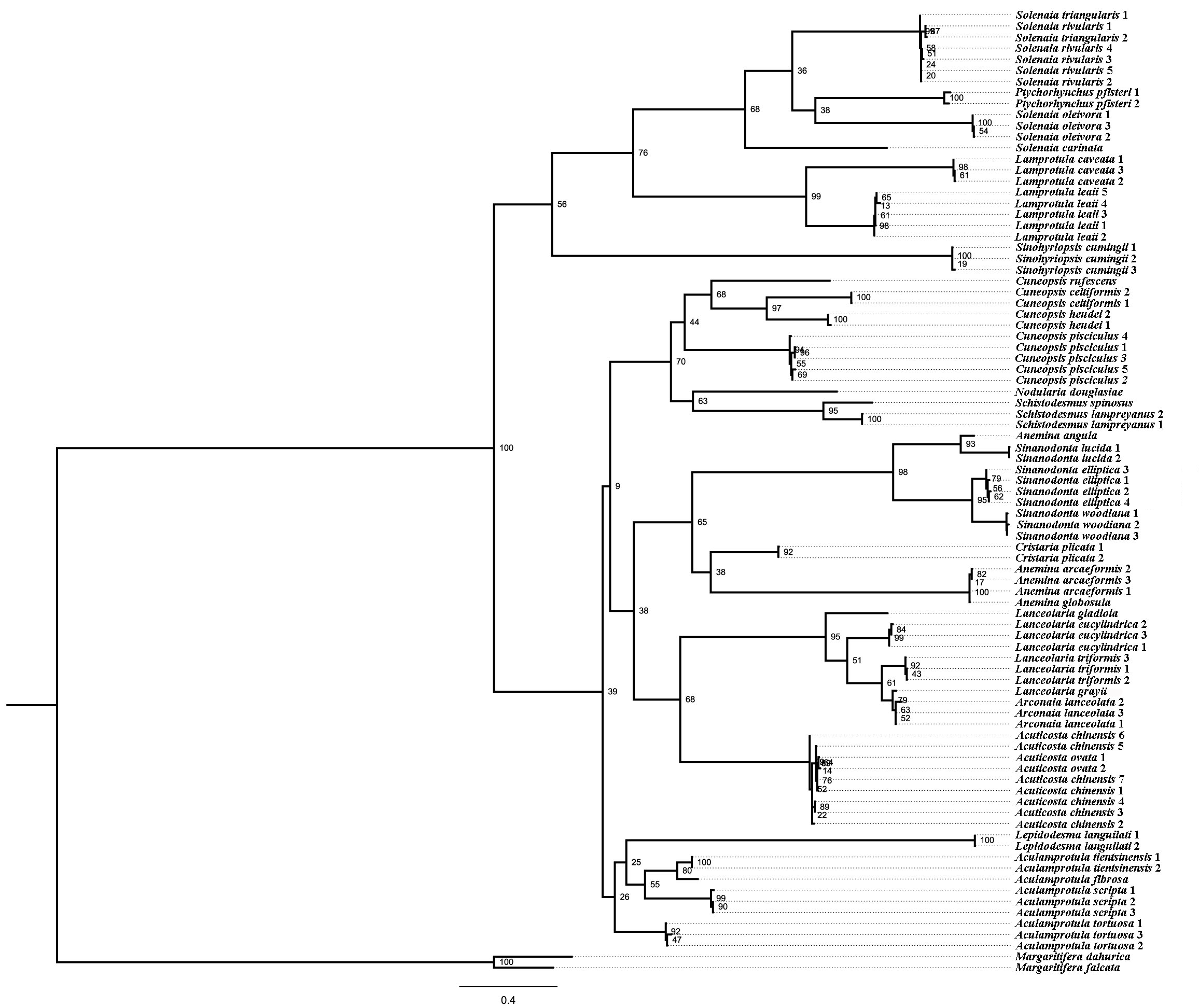

Supplement: S2 Fig — (TIF) [file pone.0200956.s004.tif]
